# Supplementary material for: Immune memory shapes human polyclonal antibody responses to H2N2 vaccination
Source: Cell Rep. 2024 May 7;43(5):114171. doi: 10.1016/j.celrep.2024.114171 (PMC11156625; doi:10.1016/j.celrep.2024.114171)
Supplement: Document S2. Article plus supplemental information [file mmc2.pdf]

Cell Reports

Article

Immune memory shapes human polyclonal antibody responses to H2N2 vaccination

Graphical abstract

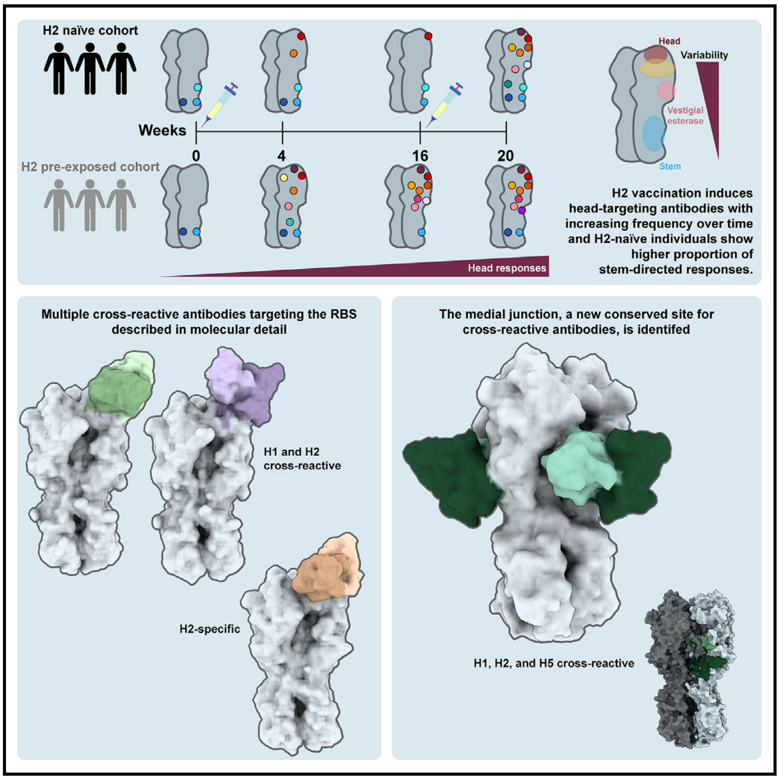

Authors

Yuhe R. Yang, Julianna Han, Hailee R. Perrett, ..., Adrian B. McDermott, Sarah F. Andrews, Andrew B. Ward

Correspondence

sarah.andrews2@nih.gov (S.F.A.), andrew@scripps.edu (A.B.W.)

In brief

Yang et al. structurally characterize antibody responses to H2N2 vaccination in H2-naive and H2-exposed people, demonstrating temporal immune biases to conserved and variable regions on HA. H2N2 vaccination elicited antibodies to conserved sites on the HA stem, receptor-binding site, and medial junction.

Highlights

- Variable HA head epitopes targeted in H2-exposed donors after H2-F vaccination
- Conserved head and stem epitopes targeted in H2-naive donors after H2-F vaccination
- RBS-targeting VH1-69 cross-reactive antibodies induced in H2-naive individuals
- The medial junction is a previously uncharacterized conserved epitope on HA

Yang et al., 2024, Cell Reports *43*, 114171
May 28, 2024 © 2024 The Authors. Published by Elsevier Inc.
https://doi.org/10.1016/j.celrep.2024.114171

CellPress

Cell Reports

CellPress
OPEN ACCESS

Article

Immune memory shapes human polyclonal antibody responses to H2N2 vaccination

Yuhe R. Yang,[1,2,4] Julianna Han,[1,4] Hailee R. Perrett,[1,4] Sara T. Richey,[1] Alesandra J. Rodriguez,[1] Abigail M. Jackson,[1] Rebecca A. Gillespie,[3] Sarah O'Connell,[3] Julie E. Raab,[3] Lauren Y. Cominsky,[3] Ankita Chopde,[3] Masaru Kanekiyo,[3] Katherine V. Houser,[3] Grace L. Chen,[3] Adrian B. McDermott,[3] Sarah F. Andrews,[3,4,*] and Andrew B. Ward[1,5,*]

[1]Department of Integrative Structural and Computational Biology, The Scripps Research Institute, La Jolla, CA 92037, USA

[2]Chinese Academy of Sciences Key Laboratory of Nanosystem and Hierarchical Fabrication, National Center for Nanoscience and Technology, Beijing 100190, China

[3]Vaccine Research Center, National Institute of Allergy and Infectious Diseases, National Institutes of Health, Bethesda, MD 20902, USA

[4]These authors contributed equally

[5]Lead contact

*Correspondence: sarah.andrews2@nih.gov (S.F.A.), andrew@scripps.edu (A.B.W.)

https://doi.org/10.1016/j.celrep.2024.114171

SUMMARY

Influenza A virus subtype H2N2, which caused the 1957 influenza pandemic, remains a global threat. A recent phase 1 clinical trial investigating a ferritin nanoparticle vaccine displaying H2 hemagglutinin (HA) in H2-naive and H2-exposed adults enabled us to perform comprehensive structural and biochemical characterization of immune memory on the breadth and diversity of the polyclonal serum antibody response elicited. We temporally map the epitopes targeted by serum antibodies after vaccine prime and boost, revealing that previous H2 exposure results in higher responses to the variable HA head domain. In contrast, initial responses in H2-naive participants are dominated by antibodies targeting conserved epitopes. We use cryoelectron microscopy and monoclonal B cell isolation to describe the molecular details of cross-reactive antibodies targeting conserved epitopes on the HA head, including the receptor-binding site and a new site of vulnerability deemed the medial junction. Our findings accentuate the impact of pre-existing influenza exposure on serum antibody responses post-vaccination.

INTRODUCTION

Responsible for causing five pandemics within the past 110 years alone, influenza viruses are one of the greatest threats to mankind. During non-pandemic years, influenza-related complications affect millions of people[1] (https://www.cdc.gov/flu/about/burden/index.html), impacting their daily lives and the global economy. Soberingly, a pandemic influenza virus is a constant threat, as the virus can undergo an antigenic shift within the vast animal reservoir and cross the species barrier,[2] as exemplified by the 1918 flu pandemic, which resulted in 50–100 million deaths. A pandemic influenza virus often features surface glycoproteins for which the human population is largely or wholly naive,[3,4] necessitating a more thorough understanding of the immune recognition of influenza subtypes by the general populace to better inform disease surveillance and pandemic prediction efforts.

Influenza A viruses are categorized by their surface glycoproteins including hemagglutinin (HA), which binds sialic acid receptors on the surface of a host cell and mediates fusion of the virus with the host endosomal membrane. Humoral immune responses to HA are known to be protective against infection by influenza[5,6] and are readily induced post-infection or post-vaccination. Yet, the success of these antibody responses—along with additional factors—drives the influenza virus to accumulate mutations to adapt its HA,[7] a process known as antigenic drift.

To prevent a future pandemic, eliciting broadly protective immunity through vaccination is our best line of defense. Antibody responses to the HA head domain are immunodominant[8] and often highly effective in neutralizing specific viral strains.[9] Yet, the head domain is highly susceptible to antigenic drift,[10–12] which enables the virus to escape these responses. Antibodies elicited by infection or vaccination that target conserved sites on HA, such as in the stem domain,[13–17] can offer protection through direct neutralization of the virus or through recruitment of adaptive and innate immune defenses to sites of infection. Various vaccination strategies, such as using novel influenza virus strains and chimeric or mosaic HAs, have shown promise in generating broadly cross-reactive and protective antibodies to these sites.[18–20]

A truly universal influenza vaccine must generate broadly neutralizing responses against the 18 recognized HA subtypes, especially those implicated in recent human pandemics: H1, H2, and H3. While only the H1N1 and H3N2 influenza A subtypes currently circulate in humans, the H2N2 influenza virus poses a distinct risk. H2N2 was the causative agent of the 1957 flu pandemic, which originally emerged from an avian reservoir.[21] This subtype resulted in more than one million deaths and

© 2024 The Authors. Published by Elsevier Inc.
This is an open access article under the CC BY license (http://creativecommons.org/licenses/by/4.0/).

circulated among the human population from 1957 until 1968 before being replaced by the H3N2 subtype. Yet, H2 influenza viruses continue to infect farm animals, birds, and swine.[22–24] Further, the H2N2 HA sequence is highly conserved between human and avian species, resulting in an ever-present risk of interspecies transmission of H2N2 and the potential to trigger a new influenza pandemic. This threat is amplified when considering that H2-specific immunity in humans exposed to H2N2 viruses pre-1968 has been waning.[25] Thus, a comprehensive analysis of human antibody responses and how these responses differ between age groups is crucial to gauge the effectiveness of candidate influenza virus vaccines. People born before 1968 likely have pre-existing immunity to H2N2 viruses due to childhood exposure. Conversely, younger populations born after 1968 are naive to the H2N2 subtype, having only been exposed to seasonal H1N1 and H3N2 strains.[23] These populations represent an excellent cohort to assess the vaccination strategy of expanding pre-existing antibody responses from one subtype (in this case, H1N1) to the conserved sites of another (H2N2).

A recent human phase 1 clinical trial (ClinicalTrials.gov: NCT03186781) assessed this vaccination strategy in fifty healthy human adults ranging in age from 18 to 47 and 52 to 70 using an H2 HA ferritin nanoparticle (H2-F) as the antigen.[26] Previous characterization of responses to the H2 antigen in this trial indicated that H2-naive individuals generated cross-reactive serological and B cell responses to the H2 stem.[26,27] Those with pre-existing immunity demonstrated more H2-specific serological antibody responses not targeting the H2 stem.[26,27] These results align with our previous work using electron microscopy polyclonal epitope mapping (EMPEM), which demonstrated at the structural level that novel vaccination elicits initial immune responses to conserved sites in novel and seasonal influenza vaccinations.[9,28–30]

Here, we aimed to map HA head- and stem-targeting antibody responses from this trial in greater detail than previously reported, pinpointing individual epitopes in order to evaluate the full potential of this vaccine. We use EMPEM and complementary serological analyses to map polyclonal antibody (pAb) responses in different age cohorts. We observe that H2-naive participants generate cross-reactive pAb responses to the receptor-binding site (RBS) in addition to the stem upon their initial exposure through H2 vaccination. Conversely, participants previously exposed to H2 show that secondary exposure through H2 vaccination generates diverse responses to strain-specific epitopes. We found that the H2-naive individuals likely recalled cross-reactive pAb responses from pre-existing immunity to H1N1 viruses. The molecular details of cross-reactive and strain-specific monoclonal antibodies (mAbs) isolated from H2-F-vaccinated individuals were revealed by high-resolution cryoelectron microscopy (cryo-EM). We also describe a broadly cross-reactive antibody to a previously unappreciated epitope on HA containing conserved residues in the central helix of HA2 and the vestigial esterase domain. This new "medial junction" epitope likely adds an additional layer of protection against diverse influenza viruses. Overall, this study enhances our understanding of both beneficial and detrimental immune responses by detailing homosubtypic and heterosubtypic pre-existing immunity following H2-F vaccination, thus contributing vital immunological insights for the optimization of influenza virus vaccines.

RESULTS

Vaccine-induced antibody responses in naive and pre-exposed individuals

A recent human phase 1 clinical trial (ClinicalTrials.gov: NCT03186781) investigated the safety and immunogenicity of two experimental H2N2-based (A/Singapore/1/1957) influenza vaccines: (1) VRC-FLUDNA082-00-VP, a plasmid DNA vaccine encoding full-length influenza A H2, and (2) VRC-FLUNPF081-00-VP, a ferritin nanoparticle presenting multivalent H2 ectodomains.[26,31] To test the safety and immunogenicity of these experimental vaccines, fifty human participants were vaccinated with different prime/boost strategies.[26] The participants were divided into two age groups—those born after 1968 without pre-existing H2N2 immunity and those exposed to H2N2 viruses before 1968 (Figure 1A, groups 1–2 and 3–4, respectively). In both age cohorts, one group received a primary vaccination of H2 DNA plasmid antigen followed by a secondary vaccination with H2-F, while the other group received a primary and secondary vaccine regimen of H2-F vaccine (Figure 1A, groups 1 and 3 and 2 and 4, respectively). Serum samples from 12 representative participants, 3 from each group, were collected at weeks 0, 4, 16, and 20 (week 4 post-boost; Figure 1B). Using a Meso Scale Discovery (MSD) assay, we observed increases in H2 HA-specific serum antibody titers over the course of vaccination in all 12 participants (Figure 1C).[26] These findings not only align with overall serum binding trends observed in each vaccine group during the clinical trial, they also provide matched serum binding profiles for the specific individuals examined in this study.[26]

In order to understand temporal dynamics and occupancy of epitopes targeted by serum pAbs after H2 vaccination, we used negative-stain EMPEM (nsEMPEM)[29,30] to map polyclonal immune complexes[16,26] with homologous H2 HA (A/Singapore/1/1957). Participant pAbs were digested to Fab, complexed in excess with HA, purified, and imaged. We analyzed the proportion of particles in each two-dimensional (2D) class based on the number of pAbs bound to HA. Consistent with sera antibody binding to immobilized H2 HA (Figure 1C), the epitope occupancy increased as more pAbs were elicited by vaccination, highlighting the immunogenicity of the H2 vaccines (Figure 1D). While the two groups who received the DNA primary vaccination showed a moderate increase in antibody titers and epitope occupancy after the priming immunization, all groups showed sustained and increased pAb responses after H2-F primary and/or secondary vaccination (Figures 1C and 1D).

Characterizing pAb responses to head and stem epitopes on H2 HA

Previous studies of antibody responses to influenza infection and vaccination revealed that cross-reactive HA stem responses are usually recalled upon exposure to antigenically novel influenza strains, while strain-specific head responses are recalled upon re-exposure to strains encountered previously.[8,29,32,33] Previous work with stem-specific probes demonstrated that

CellPress
OPEN ACCESS

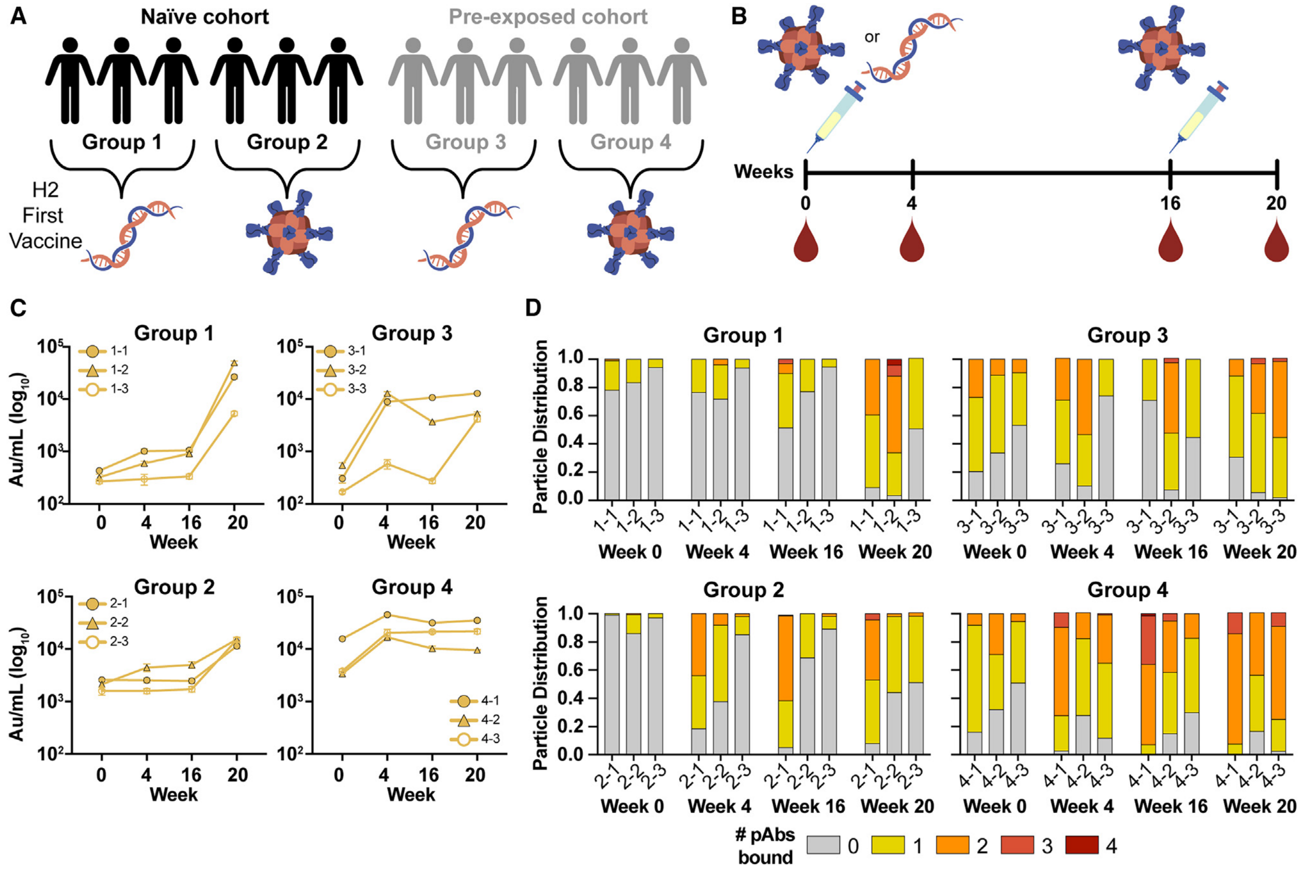

Figure 1. H2N2 vaccine elicits antigen-specific immune responses in trial participants

(A) Schematic of the H2N2 vaccine trial. Participants were placed into four groups separated by exposure status and vaccination platform: naive participants (born after H2N2 viruses ended circulation in 1968) who were primed with H2 DNA-plasmid-based vaccine (group 1) or the multivalent H2-F nanoparticle (group 2) and pre-exposed participants (born before 1968) who were first vaccinated with the DNA-plasmid-based vaccine (group 3) or the H2-F nanoparticle (group 4). All groups received secondary vaccinations with H2-F. Individual participants are notated by -1, -2, and -3 for a total of $n = 3$ per group.

(B) DNA plasmid and H2-F antigens were administered in two immunizations: first vaccine dose at week 0 and second vaccine dose at week 16. Serum samples were collected at weeks 0, 4, and 16 (after the first vaccination) and 20 (after boost).

(C) MSD binding levels of serum antibodies against H2 HA ectodomain of human participants as measured using a.u./mL (arbitrary units/mL). Serial dilutions were made and tested in duplicate, and the mean of the duplicates in the linear portion of the serial dilution curve was used to interpolate a.u./mL from the standard curve. Shown in the graph is the mean and standard deviation of the interpolated value from three dilutions in the linear portion of the curve. Data are representative of two independent experiments.

(D) nsEMPEM semi-quantitative epitope occupancy analysis denoting the proportion of HA trimers with 0, 1, 2, 3, or 4 pAbs bound (gray, yellow, orange, dark orange, and red, respectively) for each participant, noted on the x axis.

the clinical trial participants boosted cross-neutralizing stem responses upon primary exposure to H2 HA.[16,26] As differential responses within HA domains have major implications for strain-specific and cross-reactive immunity, we generated epitope landscapes of pAb responses at each time point by nsEMPEM (Figures 2 and S1–S3; Table S1). We observed clear differences in pre-existing immunity to homologous H2 HA for naive and pre-exposed groups at the serum level (Figures 2A and 2C). For the six H2-naive human participants, only stem-specific pAbs were observed at week 0, which were likely pre-existing, cross-reactive antibodies elicited by prior exposure to seasonal influenza infection or vaccination. After the first vaccination dose, pAb responses expanded to target the RBS. Responses further diversified to target variable head and vestigial esterase epitopes after the H2-F boost (Figures 2A and 2B). In contrast, the majority of pre-exposed participants had baseline pAbs targeting head and stem epitopes, which expanded after the H2-F first and/or second vaccine dose to target variable head epitopes (Figures 2C and 2D). Together, these data demonstrate that both vaccination strategies induce strong pAb responses targeting epitopes with higher sequence conservation upon primary exposure to H2 HA, while secondary exposure to H2 HA induces more diversified immunity to variable epitopes on the HA head.

While pAb maps of each time point show the diversity of epitopes targeted before and after vaccination, we hypothesized that there would be clear differences in the frequency of head versus stem responses. Thus, we analyzed overall trends of epitope distribution between serum samples using

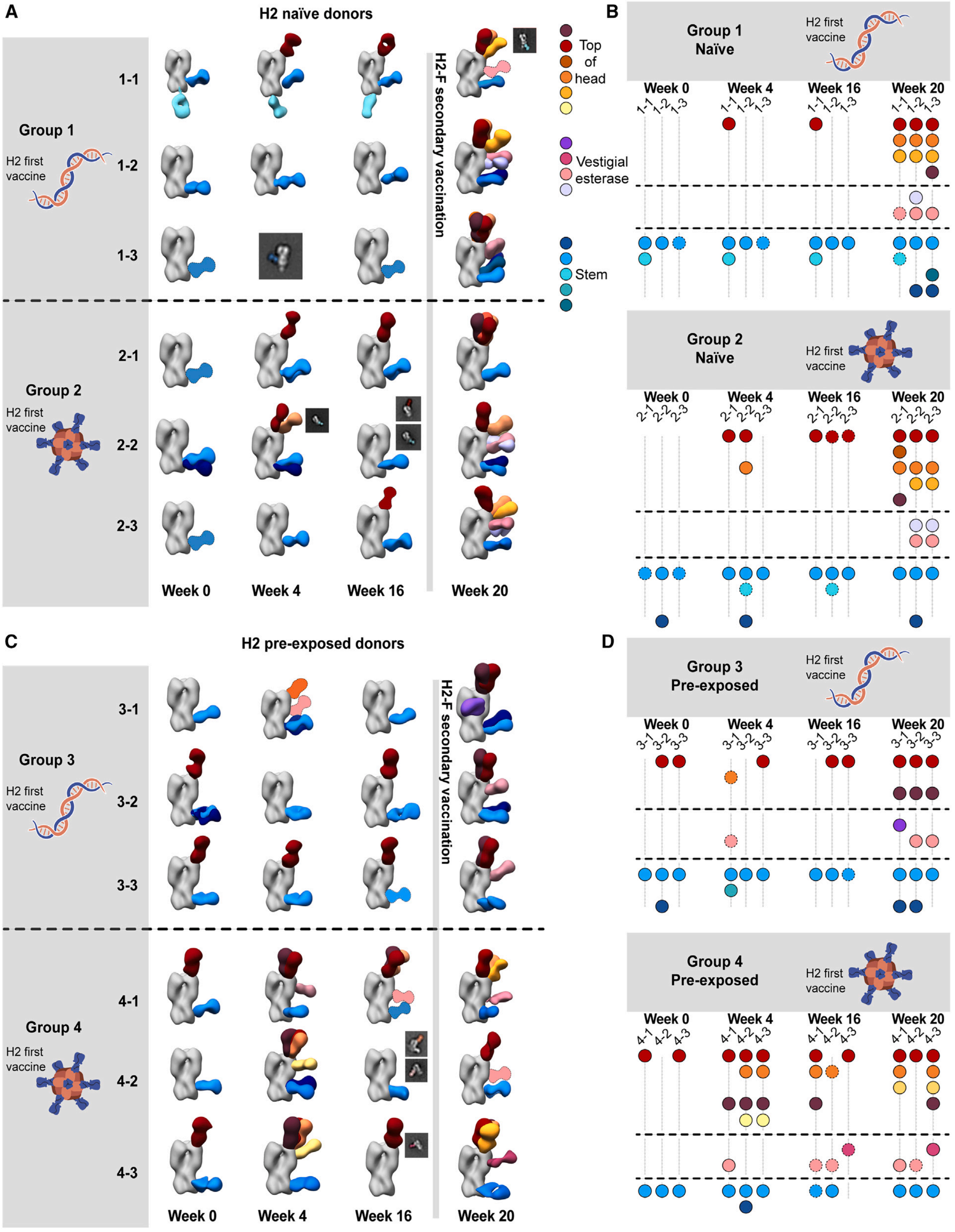

(legend on next page)

CellPress
OPEN ACCESS

semi-quantitative nsEMPEM and MSD analyses (Figure 3). Thorough 3D sorting and particle counting enabled semi-quantitative nsEMPEM analysis of the distribution of head- or stem-specific immune complexes (Figure 3A). In tandem, we performed plate-based sera binding analyses using the full-length H2 HA ectodomain (which features the head and stem) or the H2 HA stem to assess a participant's abundance of pAbs targeting the HA ectodomain (head and stem) versus the stem domain alone (Figure 3B). Both methods converged on similar trends: stem-targeting pAbs were more prominent at week 0, while head-specific antibodies dominated the pAb landscape at week 20, prompted by the H2-F boost at week 16. Overall, primary H2 vaccination with the H2-F nanoparticle boosted pAb responses to the stem, while secondary exposure, whether through the second immunization in the naive groups or through the first immunization in pre-exposed participants, elicited diverse pAb responses to head epitopes on H2 HA.

Cross-reactivity of vaccine-induced head-targeting antibodies to H1

Influenza vaccination in humans is almost always in the context of pre-existing immunity to seasonal influenza viruses. Previous work demonstrated that nearly all of the clinical trial participants had cross-reactive pre-existing immunity to stem probes from multiple HA subtypes, and H2-naive participants boosted these titers after their first H2-F immunization.[16,26] To establish a baseline of H1 head- and stem-reactive circulating antibodies and to further explore immunity elicited after H2 vaccination, we tested the ability of serum pAbs to interact with H1 HA by nsEMPEM. Evaluating H2-naive participants 1-1 and 2-2, we observed diverse pre-existing immunity to H1 with pAbs targeting epitopes on the head and stem (Figure 4A). Both donors had pre-existing immunity to the H2 HA stem epitopes and, upon vaccination, elicited responses to the HA head (Figure 4A). Neither participant had H2 head-targeting pAb responses at week 0, but both elicited responses to the RBS of H2 after H2 vaccination. In summary, pre-existing stem responses most likely elicited from prior H1 exposure can cross-react with H2 HA, while H2 HA vaccination further elicited strain-specific pAbs to the RBS.

Serum responses within an individual epitope can contain a mixed population of antibodies with varied cross-reactivity profiles. To assess epitopes targeted by cross-reactive and H2-specific head-binding antibodies, mAbs from B cells collected post-vaccination were isolated and characterized from six participants from blood collected 1 and 2 weeks after H2-F boost (Figures 4B and 4C; Table S2). nsEM epitope mapping revealed distinct patterns for each mAb group: H2-specific mAbs targeted a variety of epitopes on the HA head including RBS, vestigial esterase, and antigenic sites Sa and Cb, while the majority of cross-reactive mAbs targeted the RBS (Figure 4B). These cross-reactive RBS antibodies were detected in three subjects (1-1, 1-3, and 2-2), demonstrating that this broad antibody response is seen across individuals after H2 vaccination.

We next applied high-resolution cryo-EMPEM to a previously naive participant (1-1, week 20) to see if pAbs resembling the cross-reactive mAbs isolated from B cells in the same individual could be detected. Consistent with nsEMPEM findings (Figure 2A), cryo-EMPEM analysis of participant 1-1 revealed RBS- and stem-targeting pAbs (Figures 5A, S4C, and S4D). We obtained two high-resolution reconstructions corresponding to unique pAb complexes that targeted the RBS with distinct angles of approach (Figures 5A, S5, and S6; Table S3). We found that the pAbs overlapped with low-resolution maps of mAbs 1-1-2E05 and 1-1-1F05 isolated from participant 1-1's B cells 1–2 weeks after the H2-F boost. This suggests that these mAbs represent the corresponding antibody response within each pAb specificity (Figure 5A). For a direct comparison, we solved the structure of 1-1-1F05, which represents an abundant head-specific lineage within the B cell population (Figure 5B), in complex with H2 HA at high-resolution using cryo-EM (Figure 5C, top). Remarkably, 1-1-1F05 demonstrated high structural similarity to pAb_2 (Figure 5C, bottom). In the model of 1-1-1F05, we observed that the CDRH3 loop's interactions at the RBS epitope were in high agreement with the density map of pAb_2, strongly suggesting that 1-1-1F05 is indeed a large component of the circulating serum response to the RBS.

Structural analysis of isolated mAbs describes a binding mechanism similar to known RBS-targeting mAbs

To better understand the potential of this vaccine approach, we further dissected the differences in head-targeting antibodies (Figures 4 and 5) and compared them with known broadly neutralizing antibody (bnAb) features (Figure 6). We generated high-resolution maps of H1 cross-reactive RBS-targeting mAbs 1-1-1E04 and 1-1-1F05 as well as two H2-specific mAbs that target the RBS and antigenic site Sa (4-1-1E02 and 4-1-1G03, respectively; Figures 6A and S7). For the cross-reactive mAbs 1-1-1F05 and 1-1-1E04, we also defined their interactions with H1 (strain A/New Caledonia/20/1999(H1N1) [NC99]; Figure S8). All maps were of sufficient quality to build atomic models of HA bound to Fab except in the case of H2's cleavage site, which had heterogeneous density and was omitted from H2 models. Additionally, both H2-specific mAbs 4-1-1E02 and 4-1-1G03 were only able to neutralize the H2N2 virus (Figure S7D). Interestingly, both H1/H2 cross-reactive mAbs (1-1-1E04 and 1-1-1F05) were only able to neutralize the H1N1 virus, suggesting that additional maturation is needed to extend broad binding reactivity to broad neutralizing activity (Figure S7D).

Both cross-reactive mAbs 1-1-1F05 and 1-1-1E04 target the RBS from a single angle of approach. Relative to one another,

Figure 2. Polyclonal analysis of H2N2 vaccine trial participants

(A and C) Composite 3D reconstructions with segmented pAb specificities of each participant displayed on one protomer of the H2 HA trimer (gray) for naive participants (A) or pre-exposed participants (C). Gray lines indicates whether samples were collected pre- or post-H2F boost at week 16. Fabs represented as 2D class averages or depicted on the H2 HA trimer as a silhouette with dotted outline have limited particle representation and/or low particle abundance, and their epitopes were consequently predicted. The epitope cluster color scheme is shown on the right.

(B and D) Summary of pAb specificities for each group. Each circle represents a unique pAb specificity denoted by the color scheme in (A) and (C).

See also Figures S1–S3 and Table S1.

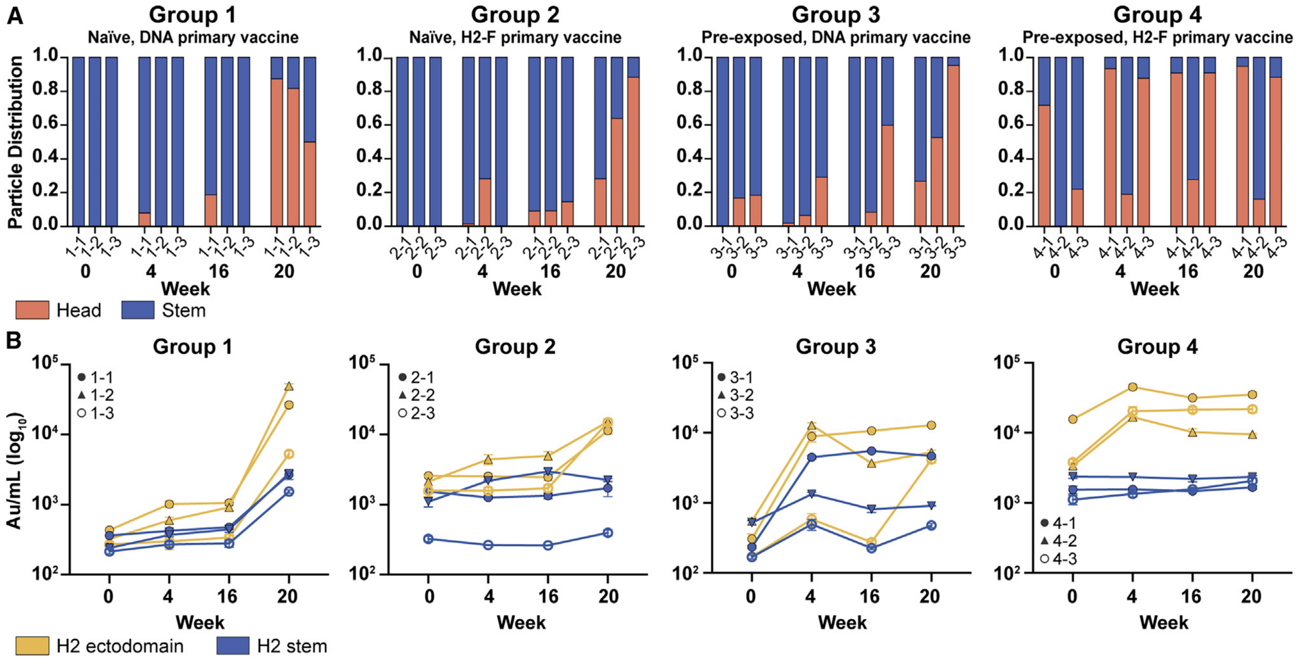

Figure 3. Frequency of H2 HA head and stem responses

(A) nsEMPEM semi-quantitative H2 HA epitope occupancy analysis indicating the proportion of pAb-containing particles in 2D classes targeting the head (orange) or stem (blue).

(B) Serum antibody titers measured by MSD using probes of HA ectodomain (gold), which includes both head and stem domains, and HA stem (blue). Serum samples of each participant are presented with unique symbols. Serial dilutions were made and tested in duplicate, and the mean of the duplicates in the linear portion of the serial dilution curve was used to interpolate a.u./mL from the standard curve. Shown in the graph is the mean and standard deviation of the interpolated value from three different dilutions in the linear portion of the curve. Data are representative of two independent experiments.

they have an approximately 90° rotation in their heavy and light chains (Figure 6A). The CDRH3 loops of these mAbs—20 residues for 1-1-1F05 and 23 for 1-1-1E04—insert into the RBS and mediate the majority of interactions with their epitopes (Figures 6B and 6C). In contrast, strain-specific mAb 4-1-1E02 had a larger epitope footprint encompassing the RBS. Its heavy and light chains mediate its interactions at the HA surface, which includes contributions from a 14-residue-long CDRH3 loop (Figures 6B and 6C). The non-RBS binding mAb 4-1-1G03 targets antigenic site Sa interacting across two protomers with contributions from its light and heavy chains (Figure S7).

As the RBS is functionally conserved, antibodies to this site have the potential to be broadly reactive.[14,34,35] We compared the H2 vaccine-elicited RBS mAbs with known neutralizing RBS mAbs to identify and dissect molecular features associated with broadly neutralizing activity. Previously, we observed the reliance of mAb 1-1-1F05's interaction with HA on its long 20 amino acid CDRH3 loop (Figure 6C, left). This interaction resembles that of bnAb C05, and structural alignment of the two reveals a near identical topology at the interface with both CDRH3 loops interacting with the RBS through a hydrogen-bonding network (Figure 6D, left). Another common feature of H2-specific neutralizing RBS mAbs is the presence of an aromatic residue that interacts with the conserved W153 of HA. This aromatic motif is found regularly in VH1-69-encoded mAbs—often as F54 in the CDRH2 loop or as another aromatic residue within the CDRH3 loop—and forms a crucial interaction with the tryptophan residing in the RBS's hydrophobic cavity.[35] mAb 1-1-1E04, which is also encoded by the VH1-69 gene, interacts in a similar manner with F100F (Kabat numbering) of its CDRH3 loop. Structural alignment of 1-1-1E04 with other VH1-69-encoded mAbs 2G1 (PDB: 4HG4), 8M2 (PDB: 4HFU), and 8F8 (PDB: 4HF5) demonstrate the similarity in aromatic interactions with HA's W153 (Figure 6D, middle). Notably, the VH4-59-encoded 1-1-1F05 lacks this signature aromatic interaction.

In contrast to the cross-reactive RBS antibodies, the H2-specific mAb 4-1-1E02, encoded by the VH4-61 gene, uses a large footprint with substantial contributions from its heavy and light chains (Figures 6C and 6D, right). Crucial interactions include P100 on its CDRH3 loop binding in the hydrophobic pocket, favorable electrostatic interactions between N33 in the CDRH1 loop and E190 of HA, and presumed hydrogen bonding between Y92 in the CDRL3 loop with N144 of HA.

Overall, H2 vaccination elicited novel antibodies that targeted a variety of epitopes including the RBS and antigenic site Sa. Moreover, multiple cross-reactive mAbs targeted the RBS with similar mechanisms to known RBS-targeting mAbs, suggesting that H2 vaccination offers a broad scope of protection by generating multiple modes of binding to the RBS.

Novel medial junction epitope targeted by H2 vaccine-elicited antibodies

Unexpectedly, in addition to RBS-targeting mAbs, we also observed cross-reactive antibody responses to non-RBS head

CellPress
OPEN ACCESS

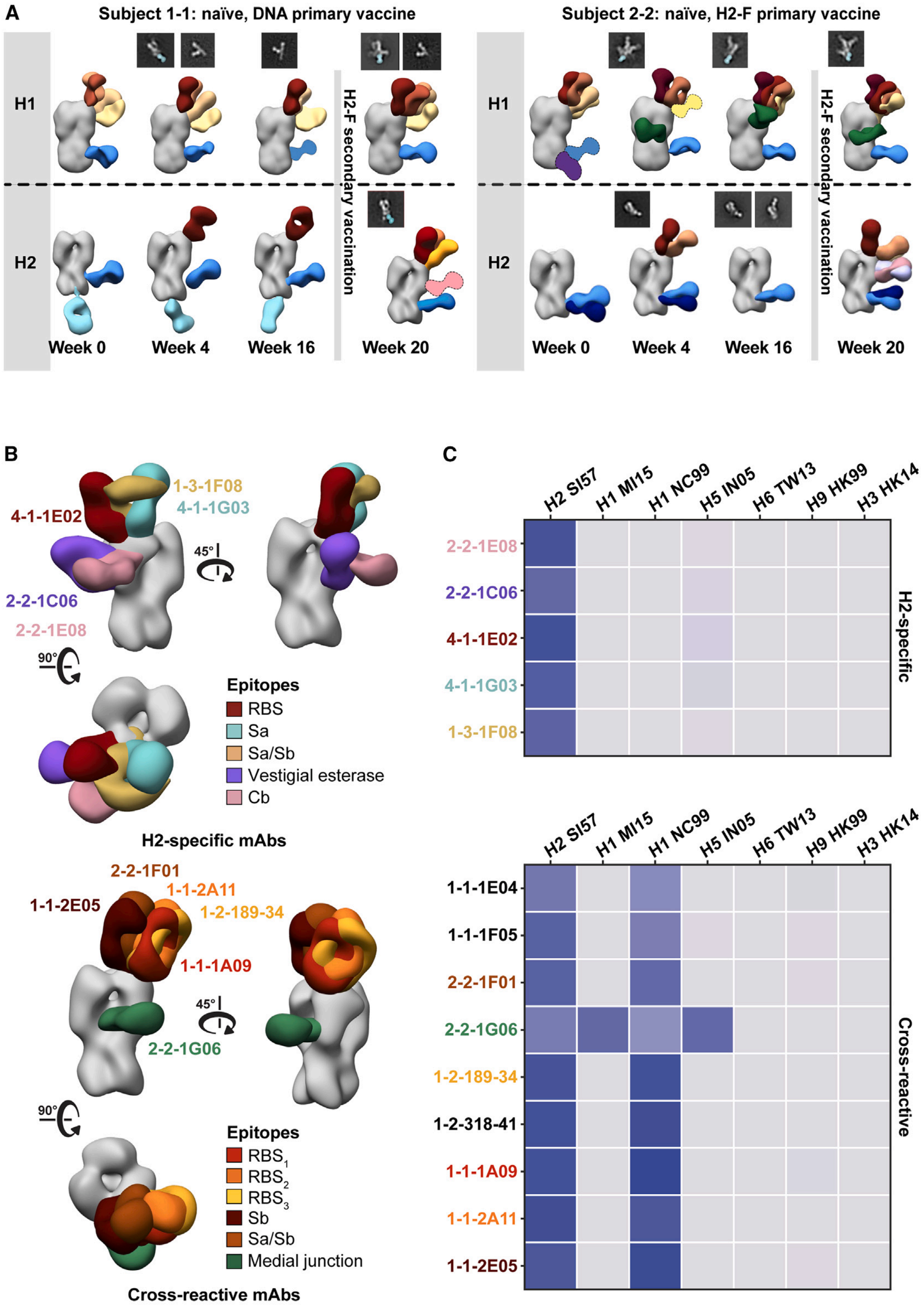

(legend on next page)

epitopes. Particularly, antibodies targeted the region between the conserved central helix and vestigial esterase domain of the HA head, termed the medial junction (Figures 4A and 4B). As a polyclonal response to the medial junction epitope was not observed by EMPEM at week 0, we expect that the pAbs binding at this epitope were boosted by H2 vaccination.

We investigated this novel epitope further by isolating cross-reactive mAb 2-2-1G06, which binds at the medial junction of H1, H2, and H5 strains (Figure 4C). While this epitope has not been previously shown to be targeted in influenza A viruses, it resembles influenza B virus bnAb CR8071.[36] To investigate the molecular interactions important for 2-2-1G06's broad reactivity, we obtained cryo-EM structures of 2-2-1G06 in complex with H2 HA (2.9 Å resolution; Figure 7A) and H1 HA (3.1 Å resolution; Figure S9A). mAb 2-2-1G06 utilizes a broad footprint with interactions from all its CDR loops to mediate binding (Figures 7B and S9B–S9E). The strongest interactions were within the CDRH3 and CDRL2 loops. Residue Y100A, which resides at the top of the CDRH3 loop, inserts into the interface area and forms a cation-π interaction with D419 of the HA's highly conserved central helix. At the turn of framework region 3, R68 faces the upper central helix of HA and interacts electrostatically with HA's E407 (Figure 7C).

The medial junction epitope is highly conserved across group 1 subtypes, as evidenced by its sequence homology among 180 sequence-aligned H2 strains (Figure 7D) and its near-universal conservation within the past 16 years for H1 viruses (Figure 7E) The epitope-paratope interface and binding topology between 2-2-1G06 and HA is structurally near identical between H1 and H2 subtypes (Figure 7F). Despite 2-2-1G06's ability to neutralize pre- and post-pandemic strains of H1 virus and cross-react to H1, H2, and H5 HA, it was unable to neutralize H2 and H5 virus strains. We expect that this may relate to 2-2-1G06's slower on rate and pronounced off rate to H2 and H5 compared to H1 (Figures 7G and S9F).

To dissect the molecular distinctions between H1 and H2 (Figure 7H) that lead to differences in 2-2-1G06 binding and neutralization, we assessed the contribution of H1's 270 loop residues, which are positioned near the apical edge of the 2-2-1G06 epitope (HA residues 265–276). We generated an H2 HA mutant with positions 265–276 mutated to H1 residues (Figure 7H; strain NC99). This mutant, named H2 S157 (270 swap), saw restored binding of 2-2-1G06, though it did not reach the potential of wild-type H1 (Figure 7I). Based on these data, we suspect that the 270 loop residues provide crucial—yet not complete—support to the overall binding potential of mAb 2-2-1G06 to H2. Overall, these studies identify an unappreciated neutralizing epitope on the medial junction of HA, which, following H2 vaccination, is targeted by antibodies with potential for broad reactivity and neutralization.

Our research shows that this H2 vaccination triggers immune responses to conserved stem epitopes, aligning with prior research,[16,26] as well as to the RBS and medial junction of the HA head domain. This multiplicity of responses broadens the protective scope of the vaccine, offering defenses across various stages of the viral life cycle. However, it is important to note that H2 vaccination can also induce responses to variable epitopes that overlap with conserved ones, leading to competition for the same antigenic site. When properly balanced, targeting multiple epitopes concurrently can be a robust strategy for developing potent vaccines that effectively guard against future viral mutations.

DISCUSSION

Human immune responses to seasonal influenza virus infection tend to be biased toward variable, strain-specific epitopes on the HA head, providing motivation to create vaccines that redirect B cell responses to conserved sites on HA. Understanding the interplay and context of antibody responses is crucial for developing vaccine regimens that boost desirable responses and limit strain-specific recall in people of different age groups. Recently, immunizations with experimental H2-F and H2 DNA plasmid vaccines were shown to induce H2-specific antibodies as well as bnAbs targeting the central stem epitope in H2-naive human populations.[26] Human clinical trial data now provide an opportunity to further refine this promising approach and advance it toward a more universal vaccine. Here, we investigated the proportion and dynamics of pAb responses to the H2 vaccines.

Our work on twelve pre-exposed and naive individuals following vaccination aligns with the observed binding profiles and stem-specific responses in the wider cohort of trial participants.[16,26] Furthermore, it extrapolates the complex interplay of antibody responses, particularly to conserved head epitopes. Primary exposure to H2 HA through vaccination with H2 DNA plasmid or H2-F vaccine candidates elicited pAb responses to conserved epitopes on HA. For H2-naive individuals, we observed through semi-quantitative nsEMPEM and serological analyses that the vast majority of H2-specific pAb responses targeted the conserved stem domain, and H1 cross-reactive RBS responses were also recalled. Moreover, secondary exposure through H2 vaccination in pre-exposed individuals or H2-F boost in naive individuals expanded the diversity of pAb responses to target variable head epitopes, shifting the dominance of pAb landscapes to the head domain. These results demonstrate the dynamics of first recalling cross-reactive memory B cells upon exposure to novel influenza virus strains while eliciting more strain-specific naive B cells, a process that amplifies upon re-exposure to the same virus.

Figure 4. Cross-reactivity of elicited immune responses

(A) Segmented nsEM 3D reconstructions of participant 1-1 (left) and 2-2 (right) pAbs complexed with either H1/NC99 or H2/1957 HA antigen. Fabs represented as 2D class averages or depicted on the H2 HA trimer as a silhouette with dotted outline have limited particle representation and/or low particle abundance, and their epitopes were consequently predicted. Gray lines indicate whether samples were collected pre- or post-H2F secondary vaccination at week 16.

(B) Representative nsEM reconstructions of H2-specific (top) and H1-cross-reactive (bottom) mAbs in complex with H2 HA.

(C) Binding levels of mAbs isolated from plasmablasts or memory B cells against HA subtypes 1 and 2 weeks after H2-F boost.

See also Figure S4 and Tables S1 and S2.

CellPress
OPEN ACCESS

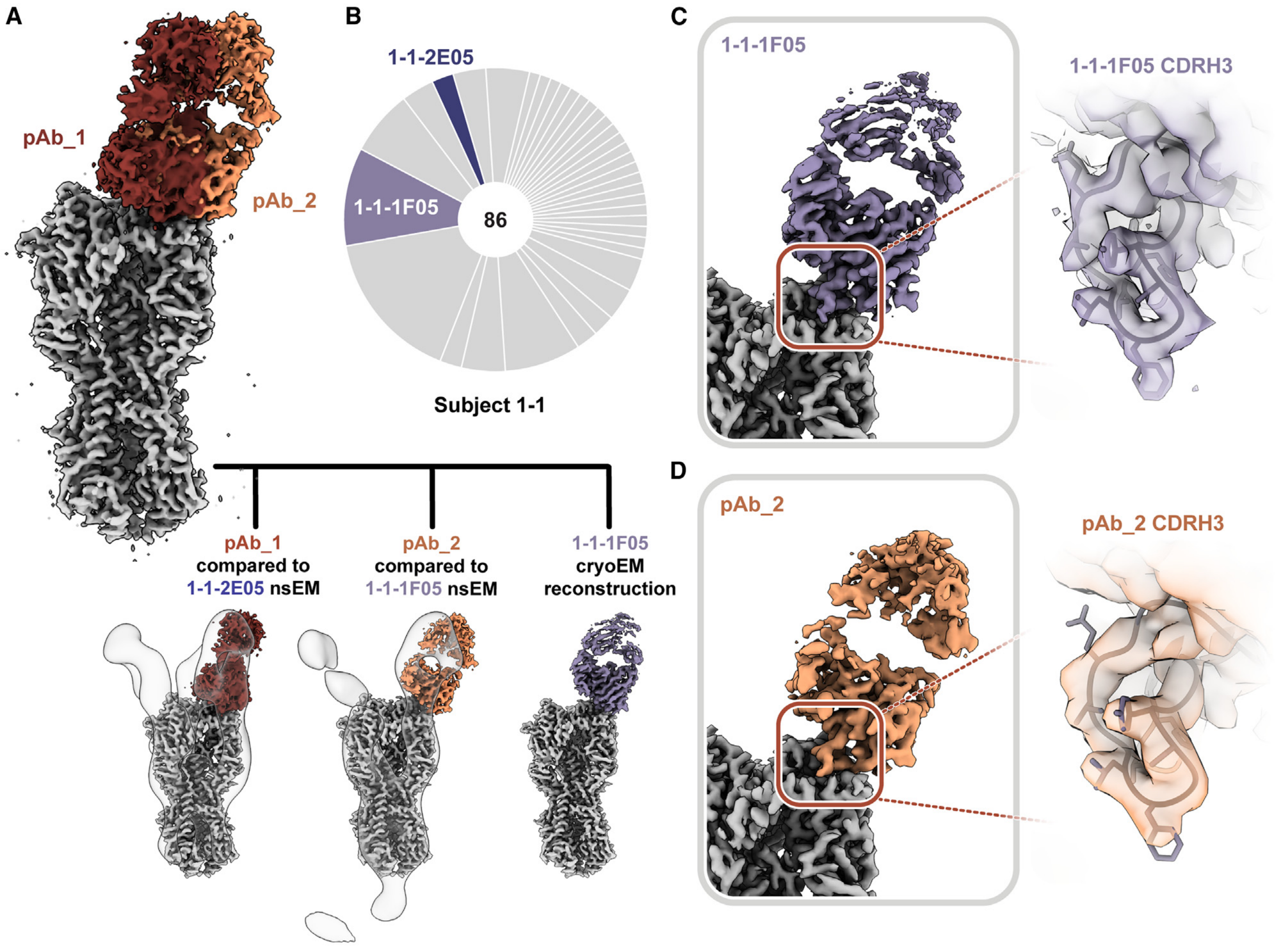

Figure 5. Structural analysis of RBS-targeting pAbs in participant 1-1

(A) Cryo-EMPEM analysis of immune complexes from participant 1-1 on week 20. H2 HA antigen is colored gray, with two segmented Fab density maps colored in red and orange (top). nsEM maps of mAbs in complex with H2 HA are overlaid against the corresponding cryo-EM map (bottom).

(B) Pie chart showing Ig repertoire of single-cell-sorted and sequenced H2 head-specific plasmablasts from participant 1-1 one week after the H2 HA ferritin boost.

(C) Single-particle cryo-EM reconstructions with zoomed-in view at the epitope-paratope interaction of H2 HA in complex with 1-1-1F05 (top) or pAb_2 (bottom). The atomic model of 1-1-1F05 is shown in purple and docked into both density maps.

See also Figures S4–S6 and Tables S2 and S3.

Quantity and quality of antibody responses can vary significantly between vaccine platforms, depending in part on immunogen display, dosing, and prime-boost intervals.[37–40] Nanoparticle-based vaccines are known to induce strong immune responses compared to typically less immunogenic modalities like DNA and subunit vaccines.[40–42] Nanoparticle and other scaffolded antigens can have increased retention in the lymph follicles, undergo complement-mediated opsonization, increase antigen-presenting cell uptake and subsequent presentation, and efficiently bind and activate multiple B cell receptors due to the multivalent nature of the presented antigen.[43–47] In line with these known differences between vaccine modalities, in earlier clinical work on this trial, researchers observed moderate immune responses after the DNA vaccine dose but observed greater surges in antibody responses after each nanoparticle administration (prime or boost).[26] They hypothesized that vaccine platform differences and timing between the DNA prime and H2-F boost may have been suboptimal for eliciting high antibody titers.

We also note stark differences between participant outcomes based on the first vaccine dose employed. Consistent with clinical data, our semi-quantitative EMPEM analyses showed the proportion of H2 HA molecules bound to pAbs increased by over 70% post-DNA plasmid primary vaccine dose (week 4) to post-H2-F boost in participant 1-1 (week 20). In general, naive participants who received an H2-F first dose showed quicker increases in pAb-bound HA (from week 0 to week 4), with less dramatic increases after H2-F boost. Regardless of first vaccine dose, H2-naive participants showed antibody responses that diversified from being almost wholly stem targeting after the primary dose to being dominated by head responses after H2-F boost. Taken together, these data demonstrate the importance

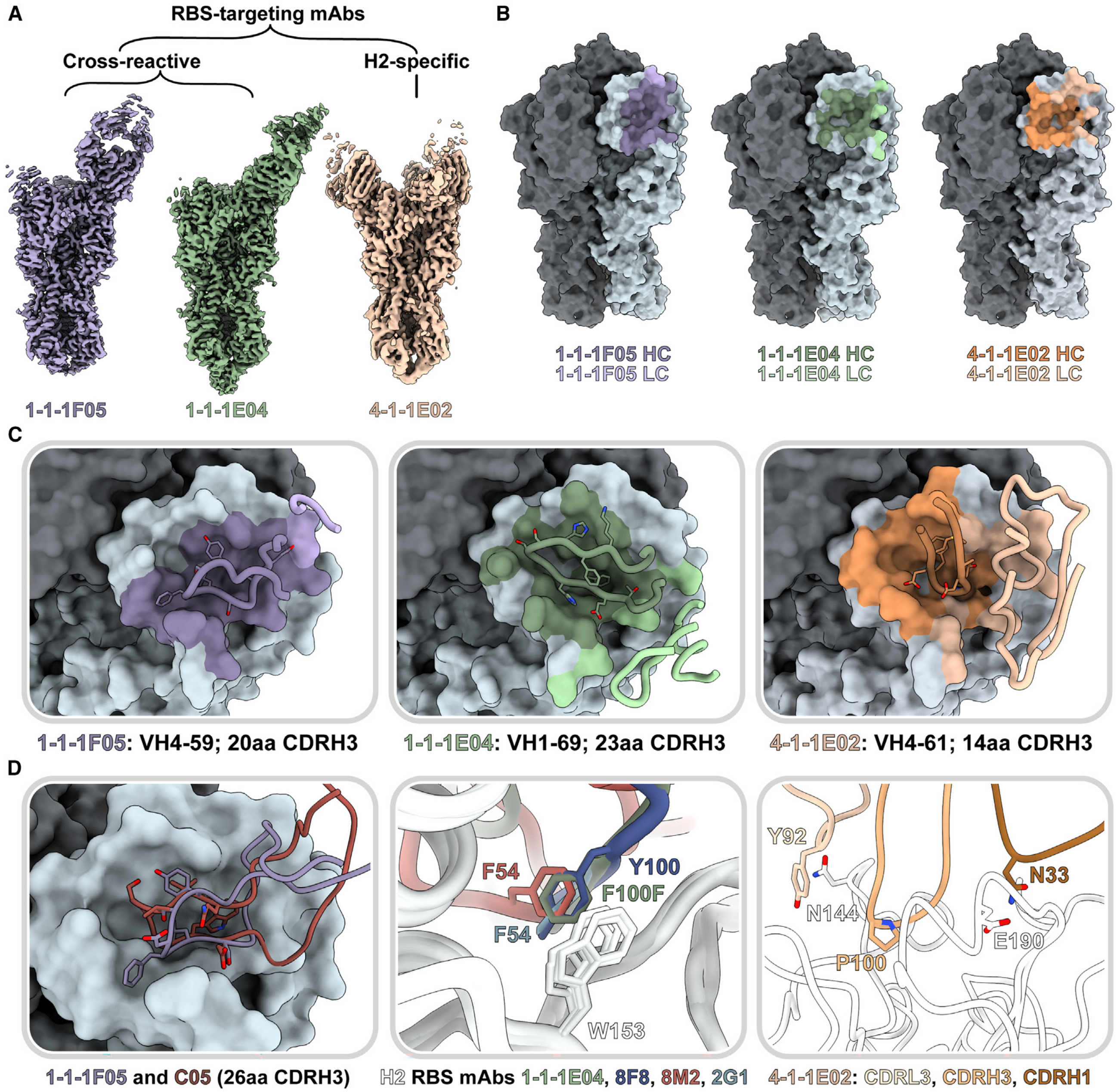

Figure 6. Structural characterization of RBS-targeting antibodies

(A) Cryo-EM density maps of mAb-HA complexes.
(B) Antibody footprints of 1-1-1F05, 1-1-1E04, and 4-1-1E02 mAbs on HA colored to indicate heavy and light chain interactions.
(C) Antibody loop interactions with the RBS pocket, with key CDRH3 residues shown. CDRH3 residue lengths are annotated using the IMGT numbering scheme.
(D) 1-1-1F05 and bnAb C05 (PDB: 4FP8) CDRH3 loops superimposed (left); 1-1-1E04 superimposed with bnAbs 2G1 (PDB: 4HF5), 8M2 (PDB: 4HFU), and 8F8 (PDB: 4HF5, middle); and 4-1-1E02 epitope-paratope interaction with key side chains shown (right).
See also Figures S5–S8 and Table S3.

of a primary vaccine dose on pAb outcomes and the polarizing phenotype of primary and secondary exposures to HA.

To improve vaccine efficacy, strategically targeting beneficial epitopes and drawing immune responses away from immunodominant, strain-specific ones is crucial. While binding assays such as ELISA can measure antigen-specific antibody responses, EMPEM is a powerful tool for mapping antibody responses to each epitope in the serum, providing immediate feedback for mechanistic evaluation of epitope-targeting in vaccines. Further, the abundance of pAbs to each epitope can be calculated to show immune transitions over time. While epitope abundance is directly affected by antibody affinity, EMPEM cannot

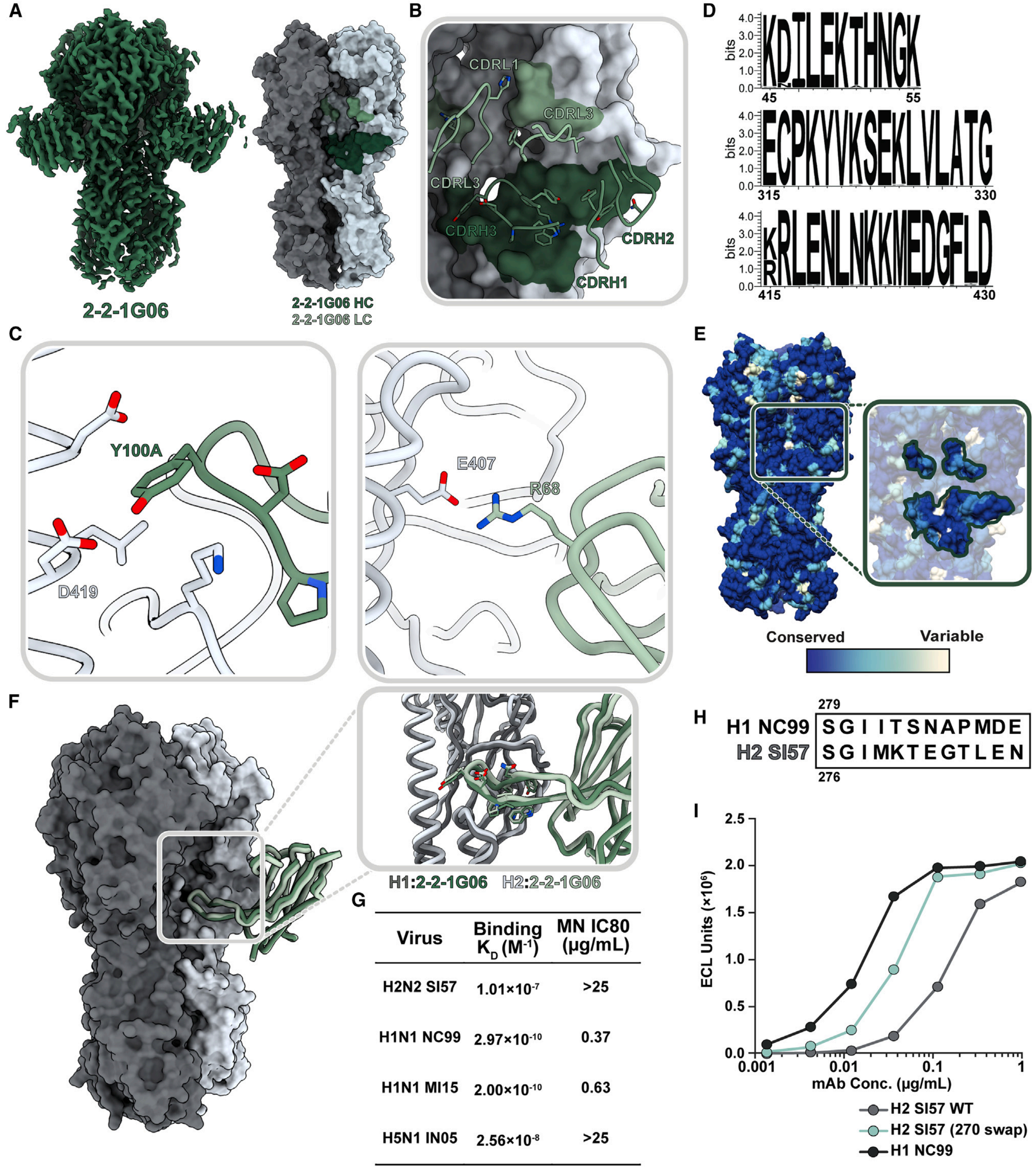

Virus	Binding K_D (M^{-1})	MN IC80 (µg/mL)
H2N2 SI57	1.01×10^{-7}	>25
H1N1 NC99	2.97×10^{-10}	0.37
H1N1 MI15	2.00×10^{-10}	0.63
H5N1 IN05	2.56×10^{-8}	>25

Figure 7. Structural and functional characterization of 2-2-1G06 targeting the novel medial junction epitope

(A) Cryo-EM map of 2-2-1G06 in complex with H2 HA (left) and antibody footprint (right).

(B) CDR loop interactions at the 2-2-1G06 epitope.

(C) 2-2-1G06 epitope-paratope interactions. Residues presumed critical for binding are shown (Y106 of the CDRH3 on the left and R68 of the CDRL2 on the right).

(D) Sequence alignment of 180 human and avian H2 viruses.

(E) 16 years of H1 HA sequence variability mapped on an HA surface. Years with sequences represented include 1999, 2006, 2007, 2008, 2009, 2011, 2013, and 2015.

(legend continued on next page)

determine the affinities or functional activity of heterogeneous antibody responses to a single epitope. Therefore, mAb analyses are a potent complement to EMPEM analyses in order to comprehensively characterize the dynamics and functionality of vaccine responses.

While the majority of universal vaccine efforts focus on the HA stem domain, conserved epitopes in the HA head domain remain promising for their ability to potently neutralize receptor binding and inhibit viral entry. The DNA plasmid and H2-F vaccines induced cross-reactive antibodies to the RBS in multiple participants that circulated as pAbs in serum. Moreover, H2 vaccination induced cross-reactive, neutralizing mAbs with diverse gene usages and mechanisms of binding, providing redundancy that may safeguard against viral escape mutations. In contrast, a strain-specific mAb elicited by secondary exposure to the vaccine had a larger footprint that extended into antigenic site Sa. These results suggest that pinpointing minimal conserved HA epitopes and key residues is crucial for expanding the breadth of Ab responses.

Novel, conserved epitopes on HA are still being discovered, such as the anchor, trimer interface, and lateral patch epitopes,[9,28,48–53] suggesting that there are more avenues to exploit for universal vaccine design. Here, we describe the novel influenza A medial junction epitope targeted by pAbs and describe its molecular details with cross-reactive mAb 2-2-1G06. This epitope resides in the cavity of the stem's central helix and head domain junction. Though this marks the first description in influenza A viruses, antibodies to a similar epitope inhibit release of progeny virions of influenza B viruses. In an H2-naive participant, we observed recall of H1-reactive medial junction cavity pAbs upon first exposure to H2. While we did not observe medial junction pAbs against H2 in serum via EMPEM, mAb 2-2-1G06 isolated from a memory B cell population of the same donor bound H1, H2, and H5 *in vitro*. Additionally, it was able to neutralize pre- and post-2009 H1N1 pandemic strains. These results suggest that H2 vaccination can induce memory B cells generated by H1N1 exposure that recall antibodies to the cross-reactive medial junction cavity in H2.

Due to the ability of influenza virus to subvert antibody responses, targeting protective epitopes and limiting person- and context-dependent variation is crucial for developing vaccines effective against diverse influenza strains regardless of exposure status. This vaccine trial provides strong proof of concept that H2 vaccination can induce cross-reactive pAbs to conserved epitopes in the RBS, medial junction, and central stem; however, we demonstrate that targeting conserved epitopes is substantially reduced if there is pre-existing immunity to H2. Our results will help inform modifications to H2 HA immunogens and prime-boost strategies to further improve vaccine efficacy and breadth regardless of exposure history.

Limitations of the study

Mapping epitopes that are targeted by serum antibodies is essential for evaluating antibody coverage of a vaccine antigen. EMPEM can generate a thorough landscape of targeted epitopes, including the relative abundance of each antibody specificity. However, EMPEM is unable to determine pAb affinities, functional activity, or cross-reactivity. Additionally, while EMPEM measures immunoglobulin G (IgG) responses in serum, it does not account for contributions from other antibody classes, such as IgA and IgM. Integrating structure-based pAb mapping with mAb analyses, functional evaluations of mAbs and serum, and cross-reactivity profiling creates a robust method for comprehensively understanding antibody responses elicited by vaccines.

STAR★METHODS

Detailed methods are provided in the online version of this paper and include the following:

- KEY RESOURCES TABLE
- RESOURCE AVAILABILITY
  - Lead contact
  - Materials availability
  - Data and code availability
- EXPERIMENTAL MODEL AND SUBJECT DETAILS
  - Cell lines
  - Selection of clinical participants
- METHOD DETAILS
  - HA expression and purification
  - Polyclonal antibody purification and digestion to pFabs
  - Monoclonal antibody digestion and Fab purification
  - HA complex formation with pFabs and monoclonal Fabs
  - Negative stain electron microscopy
  - Semi-quantitative analysis of nsEMPEM data
  - MSD binding assay
  - Single-cell sorting HA-specific B cells
  - Single-cell Ig amplification and sequencing and mAb production
  - CryoEM grid preparation and imaging
  - CryoEM data processing
  - Atomic model building and refinement
  - Sequence alignment and conservation assessment
  - Microneutralization assay
  - Biolayer interferometry

SUPPLEMENTAL INFORMATION

Supplemental information can be found online at https://doi.org/10.1016/j.celrep.2024.114171.

ACKNOWLEDGMENTS

The authors thank Bill Anderson and Hannah Turner from the Scripps Research Institute for their help with EM experiments. We thank Dr. Lauren Holden for her help in preparing this manuscript. We gratefully acknowledge all data contributors, i.e., the authors and their originating laboratories responsible for obtaining the specimens, and their submitting laboratories for generating the genetic sequence and metadata, on which this research is based,

(F) Structural comparison of 2-2-1G06 in complex with H2 and H1 NC99. Pop-out image shows CDRH3 residues.
(G) 2-2-1G06 binding affinity and microneutralization of H1, H2, and H5 viruses.
(H) 270 loop sequence alignment of H1 and H2 strains used in neutralization assay.
(I) Binding activity of 2-2-1G06 to SI57 H2 wild type (WT), H2 with H1-reverted mutations "270 swap," and H1 NC99.
See also Figures S5, S6, and S9 and Table S3.

CellPress
OPEN ACCESS

and sharing via the GISAID Initiative. This work is supported by the Bill and Melinda Gates Foundation through grant INV-004923 (to A.B.W.). J.H. is funded by NIAID 2 T32 AI007244-36. H.R.P. is supported by a David C. Fairchild Endowed Fellowship, the Achievement Rewards for College Scientists Foundation, and NIH F31 Ruth L. Kirschstein Predoctoral Award 1F31AI172358. R.A.G., S.O'C., J.E.R., L.Y.C., A.C., M.K., K.V.H., G.L.C., S.F.A., and A.B.M. are supported by funding from the NIH Intramural Research Program.

AUTHOR CONTRIBUTIONS

Conceptualization, J.H., Y.R.Y., S.F.A., A.B.W., and A.B.M.; methodology, J.H., Y.R.Y., and S.F.A.; formal analysis, Y.R.Y., J.H., S.F.A., H.R.P., S.T.R., A.M.J., A.J.R., R.A.G., S.O'C., J.E.R., L.Y.C., and A.C.; investigation, Y.R.Y., J.H., S.F.A., S.T.R., A.M.J., and A.J.R.; resources, A.B.W., A.B.M., G.L.C., and K.V.H.; writing – original draft, J.H., Y.R.Y., S.F.A., and H.R.P.; reviewing, editing, and other feedback, A.B.W. and A.B.M.; visualization, H.R.P., Y.R.Y., J.H., and S.F.A.; supervision, A.B.W., A.B.M., and M.K.; project administration, J.H. and S.F.A.; funding acquisition, A.B.W. and A.B.M.

DECLARATION OF INTERESTS

The authors declare no competing interests.

Received: August 24, 2023
Revised: March 25, 2024
Accepted: April 16, 2024
Published: May 7, 2024

REFERENCES

1. Lafond, K.E., Porter, R.M., Whaley, M.J., Suizan, Z., Ran, Z., Aleem, M.A., Thapa, B., Sar, B., Proschle, V.S., Peng, Z., et al. (2021). Global burden of influenza-associated lower respiratory tract infections and hospitalizations among adults: A systematic review and meta-analysis. PLoS Med. *18*, e1003550.
2. Cox, N.J., and Subbarao, K. (2000). GLOBAL EPIDEMIOLOGY OF INFLUENZA: Past and Present. Annu. Rev. Med. *51*, 407–421. www.annualreviews.org.
3. Palese, P. (2004). Influenza: Old and new threats. Nat. Med. *10*, S82–S87. https://doi.org/10.1038/nm1141.
4. Guan, Y., Vijaykrishna, D., Bahl, J., Zhu, H., Wang, J., and Smith, G.J.D. (2010). The emergence of pandemic influenza viruses. Protein Cell *1*, 9–13. https://doi.org/10.1007/s13238-010-0008-z.
5. Hobson, D., Curry, R.L., and Ward-Gardner, A. (1972). The Role of Serum Haemagglutination-Inhibiting Antibody in Protection against Challenge Infection with Influenza A2 and B Viruses. J. Hyg. Camb. *70*, 767–777.
6. Smith, W., Andrewes, C.H., and Laidlaw, P.P. (1935). Influenza experiments on the immunization of ferrets and mice. Br. J. Exp. Pathol. *3*, 291–302.
7. Kirkpatrick, E., Qiu, X., Wilson, P.C., Bahl, J., and Krammer, F. (2018). The influenza virus hemagglutinin head evolves faster than the stalk domain. Sci. Rep. *8*, 10432.
8. Andrews, S.F., Huang, Y., Kaur, K., Popova, L.I., Ho, I.Y., Pauli, N.T., Henry Dunand, C.J., Taylor, W.M., Lim, S., Huang, M., et al. (2015). Immune history profoundly affects broadly protective B cell responses to influenza. Sci. Transl. Med. *7*, 316ra192.
9. Guthmiller, J.J., Han, J., Li, L., Freyn, A.W., Liu, S.T.H., Stovicek, O., Stamper, C.T., Dugan, H.L., Tepora, M.E., Utset, H.A., et al. (2021). First exposure to the pandemic H1N1 virus induced broadly neutralizing antibodies targeting hemagglutinin head epitopes. Sci. Transl. Med. *13*, 1–16.
10. Huang, K.Y.A., Rijal, P., Schimanski, L., Powell, T.J., Lin, T.Y., McCauley, J.W., Daniels, R.S., and Townsend, A.R. (2015). Focused antibody response to influenza linked to antigenic drift. J. Clin. Invest. *125*, 2631–2645.
11. Anderson, C.S., Ortega, S., Chaves, F.A., Clark, A.M., Yang, H., Topham, D.J., and DeDiego, M.L. (2017). Natural and directed antigenic drift of the H1 influenza virus hemagglutinin stalk domain. Sci. Rep. *7*, 14614–14619.
12. Sandbulte, M.R., Westgeest, K.B., Gao, J., Xu, X., Klimov, A.I., Russell, C.A., Burke, D.F., Smith, D.J., Fouchier, R.A.M., and Eichelberger, M.C. (2011). Discordant antigenic drift of neuraminidase and hemagglutinin in H1N1 and H3N2 influenza viruses. Proc. Natl. Acad. Sci. USA *108*, 20748–20753.
13. Ekiert, D.C., Bhabha, G., Elsliger, M.A., Friesen, R.H.E., Jongeneelen, M., Throsby, M., Goudsmit, J., and Wilson, I.A. (2009). Antibody Recognition of a Highly Conserved Influenza Virus Epitope. Science (1979) *324*, 246–251.
14. Ekiert, D.C., Friesen, R.H.E., Bhabha, G., Kwaks, T., Jongeneelen, M., Yu, W., Ophorst, C., Cox, F., Korse, H.J., Brandenburg, B., et al. (2011). A Highly Conserved Neutralizing Epitope on Group 2 Influenza A Viruses. Science (1979) *333*, 843–850.
15. Joyce, M.G., Wheatley, A.K., Thomas, P.V., Chuang, G.Y., Soto, C., Bailer, R.T., Druz, A., Georgiev, I.S., Gillespie, R.A., Kanekiyo, M., et al. (2016). Vaccine-Induced Antibodies that Neutralize Group 1 and Group 2 Influenza A Viruses. Cell *166*, 609–623.
16. Andrews, S.F., Cominsky, L.Y., Shimberg, G.D., Gillespie, R.A., Gorman, J., Raab, J.E., Brand, J., Creanga, A., Gajjala, S.R., Narpala, S., et al. (2023). An Influenza H1 Hemagglutinin Stem-Only Immunogen Elicits a Broadly Cross-Reactive B Cell Response in Humans. Sci. Transl. Med. *15*, eade4976.
17. Widge, A.T., Hofstetter, A.R., Houser, K.V., Awan, S.F., Chen, G.L., Burgos Florez, M.C., Berkowitz, N.M., Mendoza, F., Hendel, C.S., Holman, L.A., et al. (2023). An influenza hemagglutinin stem nanoparticle vaccine induces cross-group 1 neutralizing antibodies in healthy adults. Sci. Transl. Med. *15*, eade4790.
18. Boyoglu-Barnum, S., Ellis, D., Gillespie, R.A., Hutchinson, G.B., Park, Y.J., Moin, S.M., Acton, O.J., Ravichandran, R., Murphy, M., Pettie, D., et al. (2021). Quadrivalent influenza nanoparticle vaccines induce broad protection. Nature *592*, 623–628. https://doi.org/10.1038/s41586-021-03365-x.
19. Kanekiyo, M., Joyce, M.G., Gillespie, R.A., Gallagher, J.R., Andrews, S.F., Yassine, H.M., Wheatley, A.K., Fisher, B.E., Ambrozak, D.R., Creanga, A., et al. (2019). Mosaic nanoparticle display of diverse influenza virus hemagglutinins elicits broad B cell responses. Nat. Immunol. *20*, 362–372.
20. Nachbagauer, R., Feser, J., Naficy, A., Bernstein, D.I., Guptill, J., Walter, E.B., Berlanda-Scorza, F., Stadlbauer, D., Wilson, P.C., Aydillo, T., et al. (2021). A chimeric hemagglutinin-based universal influenza virus vaccine approach induces broad and long-lasting immunity in a randomized, placebo-controlled phase I trial. Nat. Med. *27*, 106–114.
21. Shaw, M.L., and Palese, P. (2013). Orthomyxoviridae: the viruses and their replication. In Fields Virology, D.M. Knipe and P.M. Howley, eds. (Lippincott Williams & Wilkins), pp. 1647–1689.
22. Ma, W., Vincent, A.L., Gramer, M.R., Brockwell, C.B., Lager, K.M., Janke, B.H., Gauger, P.C., Patnayak, D.P., Webby, R.J., Rcicht, J.A., et al. (2007). Identification of H2N3 Influenza A Viruses from Swine in the United States. Proc. Natl. Acad. Sci. *104*, 20949–20954.
23. Jones, J.C., Baranovich, T., Marathe, B.M., Danner, A.F., Seiler, J.P., Franks, J., Govorkova, E.A., Krauss, S., and Webster, R.G. (2014). Risk Assessment of H2N2 Influenza Viruses from the Avian Reservoir. J. Virol. *88*, 1175–1188.
24. Spackman, E. (2020). Animal Influenza Virus: Methods and Protocols (Springer Protocols).
25. Nabel, G.J., Wei, C.-J., and Ledgerwood, J.E. (2011). Vaccinate for the next H2N2 pandemic now. Nature *471*, 157–158.
26. Houser, K.V., Chen, G.L., Carter, C., Crank, M.C., Nguyen, T.A., Burgos Florez, M.C., Berkowitz, N.M., Mendoza, F., Hendel, C.S., Gordon, I.J., et al. (2022). Safety and immunogenicity of a ferritin nanoparticle H2 influenza vaccine in healthy adults: a phase 1 trial. Nat. Med. *28*, 383–391.

27. Andrews, S.F., Raab, J.E., Gorman, J., Gillespie, R.A., Cheung, C.S.F., Rawi, R., Cominsky, L.Y., Boyington, J.C., Creanga, A., Shen, C.H., et al. (2022). A single residue in influenza virus H2 hemagglutinin enhances the breadth of the B cell response elicited by H2 vaccination. Nat. Med. *28*, 373–382.

28. Guthmiller, J.J., Han, J., Utset, H.A., Li, L., Lan, L.Y.L., Henry, C., Stamper, C.T., McMahon, M., O'Dell, G., Fernández-Quintero, M.L., et al. (2022). Broadly neutralizing antibodies target a haemagglutinin anchor epitope. Nature *602*, 314–320.

29. Han, J., Schmitz, A.J., Richey, S.T., Dai, Y.N., Turner, H.L., Mohammed, B.M., Fremont, D.H., Ellebedy, A.H., and Ward, A.B. (2021). Polyclonal epitope mapping reveals temporal dynamics and diversity of human antibody responses to H5N1 vaccination. Cell Rep. *34*, 108682.

30. Bianchi, M., Turner, H.L., Nogal, B., Cottrell, C.A., Oyen, D., Pauthner, M., Bastidas, R., Nedellec, R., McCoy, L.E., Wilson, I.A., et al. (2018). Electron-Microscopy-Based Epitope Mapping Defines Specificities of Polyclonal Antibodies Elicited during HIV-1 BG505 Envelope Trimer Immunization. Immunity *49*, 288–300.e8.

31. Kanekiyo, M., Wei, C.J., Yassine, H.M., McTamney, P.M., Boyington, J.C., Whittle, J.R.R., Rao, S.S., Kong, W.P., Wang, L., and Nabel, G.J. (2013). Self-assembling influenza nanoparticle vaccines elicit broadly neutralizing H1N1 antibodies. Nature *499*, 102–106.

32. Ellebedy, A.H., Krammer, F., Li, G.M., Miller, M.S., Chiu, C., Wrammert, J., Chang, C.Y., Davis, C.W., McCausland, M., Elbein, R., et al. (2014). Induction of broadly cross-reactive antibody responses to the influenza HA stem region following H5N1 vaccination in humans. Proc. Natl. Acad. Sci. USA *111*, 13133–13138.

33. Ellebedy, A.H., Nachbagauer, R., Jackson, K.J.L., Dai, Y.N., Han, J., Alsoussi, W.B., Davis, C.W., Stadlbauer, D., Rouphael, N., Chromikova, V., et al. (2020). Adjuvanted H5N1 influenza vaccine enhances both cross-reactive memory B cell and strain-specific naive B cell responses in humans. Proc. Natl. Acad. Sci. USA *117*, 17957–17964.

34. Whittle, J.R.R., Zhang, R., Khurana, S., King, L.R., Manischewitz, J., Golding, H., Dormitzer, P.R., Haynes, B.F., Walter, E.B., Moody, M.A., et al. (2011). Broadly neutralizing human antibody that recognizes the receptor-binding pocket of influenza virus hemagglutinin. Proc. Natl. Acad. Sci. USA *108*, 14216–14221.

35. Xu, R., Krause, J.C., McBride, R., Paulson, J.C., Crowe, J.E., Jr., and Wilson, I.A. (2013). A recurring motif for antibody recognition of the receptor-binding site of influenza hemagglutinin. Nat. Struct. Mol. Biol. *20*, 363–370.

36. Dreyfus, C., Laursen, N.S., Kwaks, T., Zuijdgeest, D., Khayat, R., Ekiert, D.C., Lee, J.H., Metlagel, Z., Bujny, M.V., Jongeneelen, M., et al. (2012). Highly Conserved Protective Epitopes on Influenza B Viruses. Science (1979) *337*, 1343–1348.

37. Ellis, D., Dosey, A., Boyoglu-Barnum, S., Park, Y.J., Gillespie, R., Syeda, H., Hutchinson, G.B., Tsybovsky, Y., Murphy, M., Pettie, D., et al. (2023). Antigen spacing on protein nanoparticles influences antibody responses to vaccination. Cell Rep. *42*, 113552.

38. Ledgerwood, J.E., Zephir, K., Hu, Z., Wei, C.J., Chang, L., Enama, M.E., Hendel, C.S., Sitar, S., Bailer, R.T., Koup, R.A., et al. (2013). Prime-boost interval matters: A randomized phase 1 study to identify the minimum interval necessary to observe the h5 dna influenza vaccine priming effect. J. Infect. Dis. *208*, 418–422.

39. Ledgerwood, J.E., Wei, C.J., Hu, Z., Gordon, I.J., Enama, M.E., Hendel, C.S., McTamney, P.M., Pearce, M.B., Yassine, H.M., Boyington, J.C., et al. (2011). DNA priming and influenza vaccine immunogenicity: two phase 1 open label randomised clinical trials. Lancet Infect Dis. *12*, 916–924.

40. Liu, S., Hu, M., Liu, X., Liu, X., Chen, T., Zhu, Y., Liang, T., Xiao, S., Li, P., and Ma, X. (2023). Nanoparticles and Antiviral Vaccines. Vaccines (Basel) *12*, 30.

41. Shafaati, M., Saidijam, M., Soleimani, M., Hazrati, F., Mirzaei, R., Amirheidari, B., Tanzadehpanah, H., Karampoor, S., Kazemi, S., Yavari, B., et al. (2021). A brief review on DNA vaccines in the era of COVID-19. Future Virol. *17*, 49–66. https://doi.org/10.2217/fvl-2021-0170.

42. Snapper, C.M. (2018). Distinct immunologic properties of soluble versus particulate antigens. Front. Immunol. *9*, 598. https://doi.org/10.3389/fimmu.2018.00598.

43. Jia, J., Zhang, Y., Xin, Y., Jiang, C., Yan, B., and Zhai, S. (2018). Interactions Between Nanoparticles and Dendritic Cells: From the Perspective of Cancer Immunotherapy. Front. Oncol. *8*, 404. https://doi.org/10.3389/fonc.2018.00404.

44. Bachmann, M.F., and Jennings, G.T. (2010). Vaccine delivery: A matter of size, geometry, kinetics and molecular patterns. Nat. Rev. Immunol. *10*, 787–796. https://doi.org/10.1038/nri2868.

45. Irvine, D.J., and Read, B.J. (2020). Shaping humoral immunity to vaccines through antigen-displaying nanoparticles. Curr. Opin. Immunol. *65*, 1–6. https://doi.org/10.1016/j.coi.2020.01.007.

46. Jegerlehner, A., Storni, T., Lipowsky, G., Schmid, M., Pumpens, P., and Bachmann, M.F. (2002). Regulation of IgG antibody responses by epitope density and CD21-mediated costimulation. Eur. J. Immunol. *32*, 3305–3314. https://doi.org/10.1002/1521-4141(200211)32:11<3305::AID-IMMU3305>3.0.CO;2-J.

47. Zhang, Y.N., Lazarovits, J., Poon, W., Ouyang, B., Nguyen, L.N.M., Kingston, B.R., and Chan, W.C.W. (2019). Nanoparticle Size Influences Antigen Retention and Presentation in Lymph Node Follicles for Humoral Immunity. Nano Lett. *19*, 7226–7235.

48. Benton, D.J., Nans, A., Calder, L.J., Turner, J., Neu, U., Lin, Y.P., Ketelaars, E., Kallewaard, N.L., Corti, D., Lanzavecchia, A., et al. (2018). Influenza hemagglutinin membrane anchor. Proc. Natl. Acad. Sci. USA *115*, 10112–10117. https://doi.org/10.1073/pnas.1810927115.

49. Zost, S.J., Dong, J., Gilchuk, I.M., Gilchuk, P., Thornburg, N.J., Bangaru, S., Kose, N., Finn, J.A., Bombardi, R., Soto, C., et al. (2021). Canonical features of human antibodies recognizing the influenza hemagglutinin trimer interface. J. Clin. Invest. *131*, e146791.

50. Bangaru, S., Lang, S., Schotsaert, M., Vanderven, H.A., Zhu, X., Kose, N., Bombardi, R., Finn, J.A., Kent, S.J., Gilchuk, P., et al. (2019). A Site of Vulnerability on the Influenza Virus Hemagglutinin Head Domain Trimer Interface. Cell *177*, 1136–1152.e18.

51. McCarthy, K.R., Lee, J., Watanabe, A., Kuraoka, M., Robinson-McCarthy, L.R., Georgiou, G., Kelsoe, G., and Harrison, S.C. (2021). A Prevalent Focused Human Antibody Response to the Influenza Virus Hemagglutinin Head Interface. mBio *12*, 1–11.

52. Bajic, G., Maron, M.J., Adachi, Y., Onodera, T., McCarthy, K.R., McGee, C.E., Sempowski, G.D., Takahashi, Y., Kelsoe, G., Kuraoka, M., and Schmidt, A.G. (2019). Influenza Antigen Engineering Focuses Immune Responses to a Subdominant but Broadly Protective Viral Epitope. Cell Host Microbe *25*, 827–835.e6. https://doi.org/10.1016/j.chom.2019.04.003.

53. Raymond, D.D., Bajic, G., Ferdman, J., Suphaphiphat, P., Settembre, E.C., Moody, M.A., Schmidt, A.G., and Harrison, S.C. (2017). Conserved epitope on influenza-virus hemagglutinin head defined by a vaccine-induced antibody. Proc. Natl. Acad. Sci. USA *115*, 168–173. https://doi.org/10.1073/pnas.1715471115.

54. Creanga, A., Gillespie, R.A., Fisher, B.E., Andrews, S.F., Lederhofer, J., Yap, C., Hatch, L., Stephens, T., Tsybovsky, Y., Crank, M.C., et al. (2021). A comprehensive influenza reporter virus panel for high-throughput deep profiling of neutralizing antibodies. Nat. Commun. *12*, 1722.

55. Pettersen, E.F., Goddard, T.D., Huang, C.C., Couch, G.S., Greenblatt, D.M., Meng, E.C., and Ferrin, T.E. (2004). UCSF Chimera - A visualization system for exploratory research and analysis. J. Comput. Chem. *25*, 1605–1612.

56. Pettersen, E.F., Goddard, T.D., Huang, C.C., Meng, E.C., Couch, G.S., Croll, T.I., Morris, J.H., and Ferrin, T.E. (2021). UCSF ChimeraX: Structure visualization for researchers, educators, and developers. Protein Sci. *30*, 70–82.

57. Punjani, A., Rubinstein, J.L., Fleet, D.J., and Brubaker, M.A. (2017). CryoSPARC: Algorithms for rapid unsupervised cryo-EM structure determination. Nat. Methods *14*, 290–296.
58. Suloway, C., Pulokas, J., Fellmann, D., Cheng, A., Guerra, F., Quispe, J., Stagg, S., Potter, C.S., and Carragher, B. (2005). Automated molecular microscopy: The new Leginon system. J. Struct. Biol. *151*, 41–60.
59. Zhang, K. (2016). Gctf: Real-time CTF determination and correction. J. Struct. Biol. *193*, 1–12.
60. Zivanov, J., Nakane, T., Forsberg, B.O., Kimanius, D., Hagen, W.J., Lindahl, E., and Scheres, S.H. (2018). New tools for automated high-resolution cryo-EM structure determination in RELION-3. Elife *7*, 421666–e42222.
61. Emsley, P., and Cowtan, K. (2004). Coot: Model-building tools for molecular graphics. Acta Crystallogr. D Biol. Crystallogr. *60*, 2126–2132.
62. Liebschner, D., Afonine, P.V., Baker, M.L., Bunkóczi, G., Chen, V.B., Croll, T.I., Hintze, B., Hung, L.W., Jain, S., McCoy, A.J., et al. (2019). Macromolecular structure determination using X-rays, neutrons and electrons: Recent developments in Phenix. Acta Crystallogr. D Struct. Biol. *75*, 861–877.
63. Barad, B.A., Echols, N., Wang, R.Y.R., Cheng, Y., DiMaio, F., Adams, P.D., and Fraser, J.S. (2015). EMRinger: Side chain-directed model and map validation for 3D cryo-electron microscopy. Nat. Methods *12*, 943–946.
64. Chen, V.B., Arendall, W.B., 3rd, Headd, J.J., Keedy, D.A., Immormino, R.M., Kapral, G.J., Murray, L.W., Richardson, J.S., and Richardson, D.C. (2010). MolProbity: All-atom structure validation for macromolecular crystallography. Acta Crystallogr. D Biol. Crystallogr. *66*, 12–21.
65. Lander, G.C., Stagg, S.M., Voss, N.R., Cheng, A., Fellmann, D., Pulokas, J., Yoshioka, C., Irving, C., Mulder, A., Lau, P.W., et al. (2009). Appion: An integrated, database-driven pipeline to facilitate EM image processing. J. Struct. Biol. *166*, 95–102.
66. Li, L., Changrob, S., Fu, Y., Stovicek, O., Guthmiller, J.J., McGrath, J.J.C., Dugan, H.L., Stamper, C.T., Zheng, N.Y., Huang, M., and Wilson, P.C. (2022). Librator: A platform for the optimized analysis, design, and expression of mutable influenza viral antigens. Brief. Bioinform. *23*, bbac028.
67. Voss, N.R., Yoshioka, C.K., Radermacher, M., Potter, C.S., and Carragher, B. (2009). DoG Picker and TiltPicker: Software tools to facilitate particle selection in single particle electron microscopy. J. Struct. Biol. *166*, 205–213.
68. Scheres, S. H. W. Single-particle Processing in RELION. Manuals 1–21 (2013).
69. Scheres, S.H.W. (2012). RELION: Implementation of a Bayesian approach to cryo-EM structure determination. J. Struct. Biol. *180*, 519–530.
70. Turner, J.S., Zhou, J.Q., Han, J., Schmitz, A.J., Rizk, A.A., Alsoussi, W.B., Lei, T., Amor, M., McIntire, K.M., Meade, P., et al. (2020). Human germinal centres engage memory and naive B cells after influenza vaccination. Nature *586*, 127–132.
71. Tiller, T., Meffre, E., Yurasov, S., Tsuiji, M., Nussenzweig, M.C., and Wardemann, H. (2008). Efficient generation of monoclonal antibodies from single human B cells by single cell RT-PCR and expression vector cloning. J. Immunol. Methods *329*, 112–124.
72. Doria-Rose, N.A., Schramm, C.A., Gorman, J., Moore, P.L., Bhiman, J.N., DeKosky, B.J., Ernandes, M.J., Georgiev, I.S., Kim, H.J., Pancera, M., et al. (2014). Developmental pathway for potent V1V2-directed HIV-neutralizing antibodies. Nature *509*, 55–62.
73. Zheng, S.Q., Palovcak, E., Armache, J.P., Verba, K.A., Cheng, Y., and Agard, D.A. (2017). MotionCor2: Anisotropic correction of beam-induced motion for improved cryo-electron microscopy. Nat. Methods *14*, 331–332.
74. Punjani, A., and Fleet, D.J. (2021). 3D variability analysis: Resolving continuous flexibility and discrete heterogeneity from single particle cryo-EM. J. Struct. Biol. *213*, 107702.
75. Leem, J., Dunbar, J., Georges, G., Shi, J., and Deane, C.M. (2016). ABodyBuilder: Automated antibody structure prediction with data–driven accuracy estimation. mAbs *8*, 1259–1268.
76. Weitzner, B.D., Jeliazkov, J.R., Lyskov, S., Marze, N., Kuroda, D., Frick, R., Adolf-Bryfogle, J., Biswas, N., Dunbrack, R.L., Jr., and Gray, J.J. (2017). Modeling and docking of antibody structures with Rosetta. Nat. Protoc. *12*, 401–416.
77. Zhang, Y., Aevermann, B.D., Anderson, T.K., Burke, D.F., Dauphin, G., Gu, Z., He, S., Kumar, S., Larsen, C.N., Lee, A.J., et al. (2017). Influenza Research Database: An integrated bioinformatics resource for influenza virus research. Nucleic Acids Res. *45*, D466–D474.
78. Crooks, G.E., Hon, G., Chandonia, J.M., and Brenner, S.E. (2004). WebLogo: A sequence logo generator. Genome Res. *14*, 1188–1190.

STAR★METHODS

KEY RESOURCES TABLE

REAGENT or RESOURCE	SOURCE	IDENTIFIER
Antibodies		
2-2-1C06	This paper	GenBank: PP616721, PP616735
2-2-1E08	This paper	GenBank: PP616722, PP616736
4-1-1E02	This paper	GenBank: PP616723, PP616737
4-1-1G03	This paper	GenBank: PP616724, PP616738
1-3-1F08	This paper	GenBank: PP616725, PP616739
1-2-189-34	This paper	GenBank: PP616726, PP616740
1-2-318-41	This paper	GenBank: PP616727, PP616741
1-1-1A09	This paper	GenBank: PP616728, PP616742
1-1-2A11	This paper	GenBank: PP616729, PP616743
1-1-2E05	This paper	GenBank: PP616730, PP616744
1-1-1E04	This paper	GenBank: PP616731, PP616745
1-1-1F05	This paper	GenBank: PP616732, PP616746
2-2-1F01	This paper	GenBank: PP616733, PP616747
2-2-1G06	This paper	GenBank: PP616734, PP616748
53-1F12	In house	N/A
IgG Sulfotag	MSD	Cat#R32AJ; RRID: AB_2905663
IgG+M+A: custom sulfo-tag conjugation of unlabeled	Thermo Fisher	Cat# 31128; RRID: AB_228255
CD3 BV510	BioLegend	RRID:AB_2561376
CD56 BV510	BioLegend	RRID:AB_2561385
CD14 BV510	BioLegend	RRID:AB_2561379
CD27 BV605	BioLegend	RRID:AB_11204431
CD20 APC-Cy7	BioLegend	RRID:AB_314261
IgG BV421	BD Biosciences	RRID:AB_2737665
IgM PercpCy55	BD Biosciences	RRID:AB_10611998
CD19 ECD	BD Biosciences	RRID:AB_130854
CD21 PeCy5	BD Biosciences	RRID:AB_394028
CD38	BD Biosciences	RRID:AB_1727472
Bacterial and virus strains		
R3ΔPB1 H1N1 replication-restricted reporter virus	In house (Creanga et al.)[54]	N/A
R4ΔPB1 H2N2 replication-restricted reporter virus	In house (Creanga et al.)[54]	N/A
Biological samples		
Participant serum samples	Clinical trial ID: NCT03186781	N/A
Chemicals, peptides, and recombinant proteins		
HA ectodomain from A/NewCaledonia/20/1999 H1N1	This paper	N/A
HA ectodomain from A/Michigan/45/2015 H1N1	This paper	N/A
HA ectodomain from A/Indonesia/05/2005 (H5N1)	This paper	N/A
HA ectodomain from A/Singapore/2/1957 (H2N2)	This paper	N/A
HA ectodomain from A/Singapore/2/1957 (H2N2) with 270 swap	This paper	N/A
HA stabilized stem from A/Singapore/2/1957 (H2N2)	This paper	N/A
HA ectodomain from A/Taiwan/1/2013 (H7N9)	This paper	N/A
HA ectodomain from A/Hongkong/1073/1999 (H9N2)	This paper	N/A
HA ectodomain from A/Hongkong/4801/2014	This paper	N/A
PEI MAX	Polysciences	Cat# 24765-1
PBS	Thermo Scientific	Cat# 10010023

(*Continued on next page*)

CellPress
OPEN ACCESS

Continued

REAGENT or RESOURCE	SOURCE	IDENTIFIER
TBS	Alfa Aesar	Cat# J60764.K2
Glycine	VWR	M103-1KG
Papain from papaya latex	Sigma-Aldrich	SKU# P4762
Sodium phosphate	VWR	71505-1KG
EDTA	VWR	0245-1KG
L-cysteine	Sigma	168149-100G
Iodacetamide	Sigma-Aldrich	Cat# I1149
Uranyl Formate	Electron Microscopy Sciences	Cat #D310 25 GM
Tween 20	Sigma-Aldrich	Cat# P1379-500ML
MSD Blocker A	MSD	Cat# R93BA
MSD Read Buffer	MSD	Cat# R92TC
Aqua dead cell stain	Thermo Scientific	Cat# L34957
Expifectamine	Thermo Scientifc	Cat# A14635
Expi293 Enhancer 1	Thermo Scientifc	Cat# A14635
Expi293 Enhancer 2	Thermo Scientifc	Cat# A14635
Octyl-beta-glucoside	Anatrace	Cat# O311
OptiMEM	Thermo Scientific	Cat# 31985070
TPCK treated trypsin	Thermo Scientific	Cat# 20233
Bovine Serum Albumin (fraction V)	Thermo Scientific	Cat# 9048-46-8
**Deposited data**		
Polyclonal electron microscopy maps	This paper	EMDB: EMD-41514 to EMD-41564
Monoclonal electron microscopy maps	This paper	EMDB: EMD-41683 to EMD-41694
1-1-1F05 bound to H2	This paper	PDB 8TP2; EMDB: EMD-41464
1-1-1F05 bound to H1	This paper	PDB 8TP3; EMDB: EMD-41465
1-1-1E04 bound to H2	This paper	PDB 8TP4; EMDB: EMD-41466
1-1-1E04 bound to H1	This paper	PDB 8TP5; EMDB: EMD-41467
4-1-1E02 bound to H2	This paper	PDB 8TP6; EMDB: EMD-41468
4-1-1G03 bound to H2	This paper	PDB 8TP7; EMDB: EMD-41469
2-2-1G06 bound to H2	This paper	PDB 8TP9; EMDB: EMD-41470
2-2-1G06 bound to H1	This paper	PDB 8TPA; EMDB EMD-41471
pAb_1 bound to H2	This paper	EMDB: EMD-41472
pAb_2 bound to H2	This paper	EMDB: EMD-41473
pAb_3 bound to H2	This paper	EMDB: EMD-41474
2-2-1C06 monoclonal antibody	This paper	GenBank: PP616721, PP616735
2-2-1E08 monoclonal antibody	This paper	GenBank: PP616722, PP616736
4-1-1E02 monoclonal antibody	This paper	GenBank: PP616723, PP616737
4-1-1G03 monoclonal antibody	This paper	GenBank: PP616724, PP616738
1-3-1F08 monoclonal antibody	This paper	GenBank: PP616725, PP616739
1-2-189-34 monoclonal antibody	This paper	GenBank: PP616726, PP616740
1-2-318-41 monoclonal antibody	This paper	GenBank: PP616727, PP616741
1-1-1A09 monoclonal antibody	This paper	GenBank: PP616728, PP616742
1-1-2A11 monoclonal antibody	This paper	GenBank: PP616729, PP616743
1-1-2E05 monoclonal antibody	This paper	GenBank: PP616730, PP616744
1-1-1E04 monoclonal antibody	This paper	GenBank: PP616731, PP616745
1-1-1F05 monoclonal antibody	This paper	GenBank: PP616732, PP616746
2-2-1F01 monoclonal antibody	This paper	GenBank: PP616733, PP616747
2-2-1G06 monoclonal antibody	This paper	GenBank: PP616734, PP616748

(*Continued on next page*)

Continued		
REAGENT or RESOURCE	SOURCE	IDENTIFIER
Experimental models: Cell lines		
FreeStyle 293F cells	Thermo Scientific	Cat# R79007
Expi293 cells	Thermo Scientific	Cat# A14635
MDCK-SIAT-PB1 cells	In house (Creanga et al.)[54]	N/A
Recombinant DNA		
HA ectodomain from A/NewCaledonia/20/1999 H1N1	This paper	N/A
HA ectodomain from A/Michigan/45/2015 H1N1	This paper	N/A
HA ectodomain from A/Indonesia/05/2005 (H5N1)	This paper	N/A
HA ectodomain from A/Singapore/2/1957 (H2N2)	This paper	N/A
HA ectodomain from A/Singapore/2/1957 (H2N2) with 270 swap	This paper	N/A
HA stabilized stem from A/Singapore/2/1957 (H2N2)	This paper	N/A
HA ectodomain from A/Taiwan/1/2013 (H7N9)	This paper	N/A
HA ectodomain from A/Hongkong/1073/1999 (H9N2)	This paper	N/A
HA ectodomain from A/Hongkong/4801/2014	This paper	N/A
Software and algorithms		
GraphPad Prism v8	GraphPad	N/A
UCSF Chimera	Pettersen et al.[55]	N/A
UCSF ChimeraX	Pettersen et al.[56]	N/A
cryoSPARC.v3	Punjani et al.[57]	N/A
Leginon	Suloway et al.[58]	N/A
MotionCor2	Zheng et al.[59]	N/A
GCTF	Zhang[57]	N/A
Relion/3.0 and 3.1	Zivanov et al.[60]	N/A
ABodyBuilder	Leem et al.[61]	N/A
Coot	Emsley et al.[61]	N/A
Phenix	Liebschner et al.[62]	N/A
EMRinger	Barad et al.[63]	N/A
MolProbity	Chen et al.[64]	N/A
Appion	Lander et al.[65]	N/A
FlowJo	BD Lifesciences	N/A
Librator	Li et al.[66]	N/A
Other		
FreeStyle 293 Expression medium	Gibco/Life Tech	Cat# 12338-018
HisTrap HP 5 mL column	Cytiva	Cat# 17524801
Superdex200 10/300GL Column	GE Healthcare Life Sciences	Cat# 28990944
Amicon® Ultra-4 Centrifugal Filter Unit (50 kDA MWCO)	Millipore Sigma	SKU# UFC805008
Protein G resin	Cytiva	Cat# 17061802
CaptureSelect IgG-Fc resin	Thermo Scientific	Cat# 2942852010
Pierce spin columns	Thermo Scientific	Cat# 69705
Amicon® Ultra-4 Centrifugal Filter Unit (10 kDA MWCO)	Millipore Sigma	SKU# UFC801024
Amicon Ultra-0.5 Centrifugal Filter Unit (10 kDA MWCO)	Millipore Sigma	SKU# UFC5010BK
Amicon® Ultra-4 Centrifugal Filter Unit (30 kDA MWCO)	Millipore Sigma	SKU# UFC803024
Amicon Ultra-0.5 Centrifugal Filter Unit (30 kDa MWCO)	Millipore Sigma	SKU # UFC5030BK
Amicon® Ultra-4 Centrifugal Filter Unit (100 kDA MWCO)	Millipore Sigma	SKU# UFC810024
Amicon Ultra-0.5 Centrifugal Filter Unit (100 kDa MWCO)	Millipore Sigma	SKU# UFC5100BK
PELCO easiGlow	Ted Pella Inc.	N/A
400-mesh copper grids	Electron Microscopy Sciences	Cat# 0400-Cu
Tecnai F20 electron microscope	FEI	N/A

(*Continued on next page*)

Continued

REAGENT or RESOURCE	SOURCE	IDENTIFIER
TemCam F415 CMOS camera	TVIPS	N/A
Tecnai Spirit T12	FEI	N/A
Eagle 530 CCD 4k camera	FEI	N/A
384 well Streptavidin coated SECTOR Imager 6000 Reader Plates	MSD	Cat# L21SA
MSD SECTOR Imager	MSD	N/A
FACS Aria II	BD Biosciences	N/A
Protein A Agarose	Pierce	Cat# 20333
UltrAuFoil R 1.2/1.3 grids (400-mesh)	Electron Microscopy Services	N/A
UltrAuFoil R 2/2 grids (200-mesh)	Electron Microscopy Services	Cat# Q250AR2A
Vitrobot mark IV	Thermo Scientific	N/A
FEI Titan Krios	Thermo Scientific	N/A
K2 Summit direct electron detector camera	Gatan	N/A
Talos Arctica	Thermo Scientific	N/A
Celigo Image Cytometer	Nexcelom	N/A
Octet Red96 system	Sartorius (ForteBio)	N/A
Octet Biosensors: Streptavidin (SA)	Sartorius (ForteBio)	Cat# 18-5019

RESOURCE AVAILABILITY

Lead contact

Further information and requests for resources and reagents should be directed to and will be fulfilled by the lead contact, Andrew B. Ward (andrew@scripps.edu).

Materials availability

All reagents will be made available on request after completion of a Materials Transfer Agreement.

Data and code availability

- Maps generated from the electron microscopy data are deposited in the Electron Microscopy Databank (http://www.emdatabank.org/) under accession IDs EMDB: EMD-41464, EMD-41465 EMD-41466 EMD-41467 EMD-41468 EMD-41469 EMD-41470 EMD-41471 EMD-41472 EMD-41473 EMD-41474, EMD-41514, EMD-41515, EMD-41516, EMD-41517, EMD-41518, EMD-41519, EMD-41520, EMD-41521, EMD-41522, EMD-41523, EMD-41524, EMD-41525, EMD-41526, EMD-41527, EMD-41528, EMD-41529, EMD-41530, EMD-41531, EMD-41532, EMD-41533, EMD-41534, EMD-41535, EMD-41536, EMD-41537, EMD-41538, EMD-41539, EMD-41540, EMD-41541, EMD-41542, EMD-41543, EMD-41544, EMD-41545, EMD-41546, EMD-41547, EMD-41548, EMD-41549, EMD-41550, EMD-41551, EMD-41552, EMD-41553, EMD-41554, EMD-41555, EMD-41556, EMD-41557, EMD-41558, EMD-41559, EMD-41560, EMD-41561, EMD-41562, EMD-41563, EMD-41564, EMD-41683, EMD-41684, EMD-41685, EMD-41686, EMD-41687, EMD-41688, EMD-41689, EMD-41690, EMD-41691, EMD-41692, EMD-41693, and EMD-41694. See Tables S1–S3, for more details. Atomic models corresponding to these maps have been deposited in the Protein DataBank (http://www.rcsb.org/) under accession IDs PDB: 8TP2, 8TP3, 8TP4, 8TP5, 8TP6, 8TP7, 8TP9, and 8TPA. Monoclonal antibody sequences are deposited in GenBank under accession IDs GenBank: PP616721-PP616748.
- This paper does not report original code.
- Any additional information required to reanalyze the data reported in this work is available from the lead contact upon request.

EXPERIMENTAL MODEL AND SUBJECT DETAILS

Cell lines

HEK 293F cells were purchased from ThermoFisher Scientific. The cells were used following manufacturer protocols with details described below.

Selection of clinical participants

The VRC 316 clinical trial was a phase I, open-label, and randomized (ClinicalTrials.gov, NCT03186781) and has been described previously.[26] In short, the study was conducted at the National Institutes of Health (NIH) Clinical Center by the Vaccine Research Center Clinical Trials Program of the National Institute of Allergy and Infectious Diseases (NIAID). Trial protocols were approved by the NIAID institutional review board and informed consent was obtained from each enrolled participant. The trial evaluated H2 vaccination with

H2 plasmid DNA encoding H2 A/Singapore/1/1957 or homologous H2 HA Ferritin nanoparticle followed by a boost 16 weeks later in all participants with the H2 HA Ferritin nanoparticle vaccine. For each of the two vaccine regimens, participants born before 1966 (H2 pre-exposed) or after 1969 (H2 naive) were enrolled for a total of 4 vaccine groups. Twelve representative participants, 3 from each of the trial groups were chosen for ad-hoc analysis in this study.

METHOD DETAILS

HA expression and purification

HA proteins were transiently expressed in HEK 293F cells (Thermo Fisher) at a density of 1.0 x 10^6 cells/mL with a 1:3 ratio of DNA to PEIMax. HEK 293F cells were maintained in 293FreeStyle expression medium (Life Technologies) and cultured at 37°C, 8% CO_2, and shaken at 125 rpm. Six days after transfection, cells were harvested and spun down. HAs were purified by a HisTrap column (Cytiva). After elution, HA trimers were purified by size exclusion chromatography using a Superdex 200 Increase 10/300 column (GE Healthcare). Fractions corresponding to trimeric HA were pooled, concentrated, and buffer exchanged to TBS using 50 kDa Amicon concentrators.

Polyclonal antibody purification and digestion to pFabs

Serum samples were collected from participants in the H2 vaccine clinical trial (NCT03186781).[26] Serum samples were first heat inactivated in a 55°C water bath for 30 min. Inactivated serum was incubated for 20 h with protein G (GE Healthcare) or CaptureSelect resin (Thermo Fisher) at a ratio of 1 mL serum to 1 mL resin slurry. Samples were centrifuged briefly and IgG-depleted serum removed. The IgG-rich resin was washed three times with PBS by centrifugation. IgG was eluted from resin after incubating with 0.1 M glycine, pH 2.5 buffer for 20 min. The eluent was then neutralized with a 1 M Tris-HCl pH 8.0 buffer. The solution was buffer exchanged to PBS using 50 kDa Amicon concentrators.

Next, purified IgGs were digested to pFabs. Papain (Sigma Aldrich) was activated in fresh digestion buffer (20 mM sodium phosphate, 10 mM EDTA, 20 mM cysteine at pH 7.4). We incubated 4 mg of polyclonal IgG with activated, immobilized papain for 18–22 h at 37°C. Digested IgG was separated from papain using Pierce spin columns (Thermo Fisher) and buffer exchanged to TBS using 50 kDa Amicon concentrators. We separated pFab and Fc from undigested IgG using size exclusion chromatography with a Superdex 200 Increase 10/300 column (GE Healthcare) and concentrated the pFab/Fc.

Monoclonal antibody digestion and Fab purification

Fabs of monoclonal antibodies were generated by papain digestion of purified IgG. Papaya latex papain (Sigma Aldrich) was activated in a fresh solution of 20 mM sodium phosphate, 10 mM EDTA, and 20 mM cysteine at pH 7.4 for 15 min at 37°C. IgG was digested in the activated papain solution for 4 h at 37°C in a ratio of 1 mg IgG to 40 μg papain. The reaction was quenched using 50 mM iodoacetamide. The digestion products were buffer exchanged to PBS via centrifugation with 30 kDa Amicon concentrators, purified on a Superdex 200 Increase 10/300 column (GE Healthcare), and concentrated using 30 kDa Amicon concentrators.

HA complex formation with pFabs and monoclonal Fabs

PFab-HA complexes were obtained by incubating 500 μg concentrated pFab/Fc mixture with 10 μg recombinant HA at room temperature for 16–20 h. Complexes were purified from unbound HA, pFab, and Fc using size exclusion chromatragraphy with a Superdex 200 Increase 10/300 column (GE Healthcare) and concentrated using 100 kDa Amicon concentrators. Monoclonal Fabs were incubated with HA at a 3:1 M ratio for 1 h at room temperature.

Negative stain electron microscopy

Immune complexes were deposited on glow-discharged (PELCO easiGlow, Ted Pella, Inc.) carbon-coated 400 mesh copper grids (Electron Microscopy Sciences) at a concentration of approximately 20 μg/mL. Excess sample was removed by blotting, and grids were stained with two back-to-back depositions of 2% w/v uranyl formate for 60 s each. Excess stain was removed by blotting and the grids were allowed to dry.

Grids were imaged on either a 200 kV Tecnai F20 electron microscope (FEI) with a TemCam F416 CMOS camera (TVIPS) or a 120 kV Tecnai Spirit T12 (FEI) with an Eagle CCD 4k camera (FEI). Images were collected at 62,000 or 52,000× magnification with pixel sizes of 1.77 and 2.06 Å, respectively. Micrographs were acquired using the Leginon software package and Appion was used to pick 100,000–400,000 single particles.[58,65,67] Particles were then processed to reference-free 2D class averages and 3D reconstructions using Relion.[60,68,69] UCSF Chimera and UCSF ChimeraX were used to analyze data and generate Figures [55,56] Due to limitations such as low particle count for rare polyclonal specificities and lack of angular sampling, some epitope specificities were not amenable to 3D reconstruction but showed clear specificity to HA epitopes as confirmed by distinct 2D class averages, as has been observed previously.[70] When applicable, these specificities are shown as flat surface colors with dotted black outlines.

Semi-quantitative analysis of nsEMPEM data

After the first round of reference-free 2D class averaging of polyclonal samples post-EM imaging, all classes with HA were selected, removing junk particles. A second round of reference-free 2D class averaging was performed on the selected particle stack. For semi-quantitative analysis, only side view particles were counted, as it is not possible to distinguish epitope specificities with top views. Classes were grouped according to number of pFabs bound to HA (0–4 pFabs/HA) and particle counts from Relion[60] were recorded for each group. As 2D classification is often inadequate to assign overlapping and neighboring epitopes, pFab specificities to the head and stem domains were also grouped and counted, rather than individual specificities. Particle counts for pFab abundance and head/stem specificity were conducted on the same particle stacks independently.

MSD binding assay

Meso Scale Discovery (MSD) 384 well Streptavidin coated SECTOR Imager 6000 Reader Plates were blocked with 5% MSD Blocker A for 30 to 60 min, then washed six times with the wash buffer (PBS+0.05% Tween). The plates were then coated with biotinylated HA protein (same protein as was used for flow cytometry) for 1 h and washed. mAbs were diluted in 1% MSD Blocker A to 1 μg/ml, serially diluted 3-fold, and added to the coated plates. Serum samples were diluted 1:100 in 1% MSD Blocker A and serially diluted 3-fold before adding to coated plates. A control mAb (53-1F12)(Andrews et al., 2017) was added to each plate to use a reference standard for each assay. After a 1 h incubation with sera or mAbs, plates were washed and incubated for 1 h with SULFO-TAG conjugated anti-human IgG for mAbs (MSD) or SULFO-TAG conjugated polyclonal anti-human IgG+A+M (Thermo Fisher) for serum samples. After washing, the plates were read using 1X MSD Read Buffer using an MSD SECTOR Imager 600. For mAbs, binding curves were plotted and the area under the curve (AUC) was determined using GraphPad Prism 8. For sera, binding of 1 μg/mL of 53-1F12 to H2 or H2 stabilized stem was assigned a concentration of 100 arbitrary units per milliliter (AU/mL). Serial dilutions of sample within the dynamic range of the standard curve were interpolated to assign a sample concentration in AU/mL. Results were plotted and analyzed using GraphPad Prism 8.

The following HA strains were used for mAb and/or serum binding assays: H1 A/NewCaledonia/20/1999 (NC99) ectodomain, H1 A/Michigan/45/2015 (MI15) ectodomain, H5 A/Indonesia/05/2005 (IN05) ectodomain, H2 A/Singapore/2/1957 (SI57) ectodomain, H2 SI57 ectodomain with 270 swap, H2 SI57 stabilized stem, H6 A/Taiwan/1/2013 (TW13) ectodomain, and H9 A/Hongkong/1073/1999 (HK99) ectodomain, and H3 A/Hongkong/4801/2014 (H3 HK14).

Single-cell sorting HA-specific B cells

Cryopreserved PBMCs from blood collected 1 and 2 weeks after the H2-F boost were stained with anti-human monoclonal antibodies CD3 BV510 (OKT3, 1:400 dilution, BioLegend, RRID:AB_2561376), CD56 BV510 (HCD56, 1:200 dilution, BioLegend, RRID:AB_2561385), CD14 BV510 (M5E2, 1:200 dilution, BioLegend, RRID:AB_2561379), CD27 BV605 (O323, 1:50, BioLegend, RRID:AB_11204431), CD20 APC-Cy7 (2H7, 1:400 dilution, BioLegend, RRID:AB_314261), IgG BV421 (G18-145, 1:50 dilution, BD Biosciences, RRID:AB_2737665), IgM PercpCy55 (G20-127, 1:40 dilution, BD Biosciences, RRID:AB_10611998), CD19 ECD (H3-119, 1:50 dilution, BD Biosciences, RRID:AB_130854), CD21 PeCy5 (B-ly4, 1:100 dilution, BD Biosciences, RRID:AB_394028) and CD38 (HIT2, 1:400 dilution, BD Biosciences, RRID:AB_1727472). H2 A/Singapore/2/1957 ectodomain and stabilized stem HA probes were expressed, biotinylated and labeled with fluorochromes as described previously (Whittle et al., 2014). Aqua dead cell stain was added for live/dead discrimination (ThermoFisher Scientific). Stained samples were run on a FACS Aria II (BD Biosciences) and data analyzed using FlowJo (TreeStar). $CD3^-$ $CD14^-$ $CD56^-$ $CD19^+$ $CD20^-$ $CD21^-$ $CD27^{hi}$ $CD38^{hi}$ plasmablasts or $CD3^-$ $CD14^-$ $CD56^-$ $CD19^+$ $CD20^+$ IgG+ IgM- Memory B cells were gated, and H2 HA-binding B cells were single-cell sorted into 96-well plates. H2 HA head-specific B cells were identified by indexing.

Single-cell Ig amplification and sequencing and mAb production

Reverse transcription was performed on sorted cells and multiplexed PCR was used to amplify immunoglobulin heavy and light chain genes as described previously.[71,72] We obtained paired heavy and light chain Ig sequences from an average of 70% of single cells on which we performed PCR. PCR products were sequenced by Beckman Coulter or Genewiz.

Heavy and light chain sequences were synthesized and cloned by Genscript into IgG1, kappa, or lambda expression vectors. To produce mAbs recombinantly, Expi293 cells were transfected with plasmids encoding Ig heavy and light chain pairs with ExpiFectamine (ThermoFisher Scientific). Monoclonal antibodies were purified from the cell supernatant using Sepharose Protein A (Pierce).

CryoEM grid preparation and imaging

Immune complexes were prepared as described above and applied to grids at a concentration of 0.4–0.8 mg/mL. Octyl-beta-glucoside detergent was added to samples at a final concentration of 0.1% immediately before deposition on glow-discharged Au 1.2/1.3 400-mesh and 2/2 200 mesh grids (Electron Microscopy Services). Samples were incubated on grids for 7 s before being blotted off and plunge-frozen in liquid ethane using a Vitrobot mark IV (Thermo Fisher).

After freezing, cryo grids were loaded into a 300 kV FEI Titan Krios or 200 kV Talos Arctica (Thermo Fisher), both of which were equipped with K2 Summit direct electron detector cameras (Gatan). Data were collected with approximate exposures of 50 $e^{-}/Å^{2}$. Magnifications of 130,000 or 36,000X were used for the Krios or Arctica, respectively. Data collection was automated using Leginon. Further details are described in Table S1.

CryoEM data processing

Image pre-processing was performed with the Appion software package.[65] Micrograph movie frames were aligned, dose-weighted using the UCSF MotionCor2 software,[73] and GCTF was estimated.[59] Micrographs were then transferred to CryoSPARC v3.0 for particle picking and reference-free 2D classification.[57] Initial 2D classes of high quality were used as templates for template picking of datasets followed by 2D classifications to remove bad particles. Global 3D refinements were performed, and particle stacks were sorted for Fab-bound complexes by heterogeneous refinements and 3D variability analyses.[74] Some datasets were further analyzed in Relion, where they were sorted using alignment-free 3D classification.

For polyclonal samples, 40 Å sphere masks were used to separate particles with pAbs bound within the masked area by 3D Variability (CryoSPARC) or through alignment-free 3D classification (Relion). Once pAb complexes were separated, they were refined separately and new masks featuring the full immune complex were used for final refinements.

More details are described in Table S1 including imposed symmetry and final particle counts. Figures were made using UCSF Chimera and ChimeraX.

Atomic model building and refinement

For monoclonal EM maps, we refined atomic models using corresponding post-processed maps. PDBs 6CF7 and 2WR7 were used as the initial H1 and H2 models, respectively. PDB 6CF7 was mutated to the A/New Caledonia/20/1999 sequence. Initial models for Fabs were predicted using ABodyBuilder.[75] Both HA and Fab models were manually fit into density using Coot.[61] Iterative manual model building in Coot followed by Rosetta[76] relaxed refinements were used to generate atomic models of each complex. We evaluated our models using MolProbity and EMRinger of the Phenix software package[62–64] and the PDB validation server. Epitope-paratope interactions were analyzed and visualized in UCSF Chimera and ChimeraX. Models are numbered based on the H3 numbering system for HA and the Kabat numbering system for Fabs.

Sequence alignment and conservation assessment

H1 HA sequence variability was assessed based on 8 distinct human H1 strains from 1999, 2006, 2007 2008, 2009, 2011, 2013, and 2015. The conservation model was generated using sequence logo in the Librator[66] application and visualized in UCSF Chimera. A survey of 180 H2 HA sequences from human and avian viruses was conducted using sequences from the Influenza Research Database[77] and represented as a sequence logo using the web logo tool.[78]

Microneutralization assay

Generation of the replication-restricted reporter (R3ΔPB1) virus H1N1 and Rewired R3ΔPB1 (R4ΔPB1) virus H2N2 has been described elsewhere.[54] Briefly, to generate the R3/R4ΔPB1 viruses the viral genomic RNA encoding functional PB1 was replaced with a gene encoding the fluorescent protein (TdKatushka2), and the R3/R4ΔPB1 viruses were rescued by reverse genetics and propagated in the complementary cell line which expresses PB1 constitutively. Each R3/R4ΔPB1 virus stock was titrated by determining the fluorescent units per mL (FU/mL) prior to use in the experiments. For virus titration, serial dilutions of virus stock in OptiMEM + TPCK were mixed with pre-washed MDCK-SIAT-PB1 cells (8 x 105 cells/ml) and incubated in a 384-well plate in quadruplicate (25 μL/well). Plates were incubated for 18–26 h at 37°C with 5% CO2 humidified atmosphere. After incubation, fluorescent cells were imaged and counted by using a Celigo Image Cytometer (Nexcelom) with a customized red filter for detecting TdKatushka2 fluorescence.

For the microneutralization assay, serial dilutions of antibody were prepared in OptiMEM and mixed with an equal volume of R3/R4ΔPB1 virus (~8 x 104 FU/mL) in OptiMEM + TPCK. After incubation at 37°C and 5% CO2 humidified atmosphere for 1 h, pre-washed MDCK-SIAT-PB1 cells (8 x 105 cells/well) were added to the serum-virus mixtures and transferred to 384-well plates in quadruplicate (25 μL/well). Plates were incubated and counted as described above. Target virus control range for this assay is 500 to 2,000 FU per well, and cell-only control is acceptable up to 30 FU per well. The percent neutralization was calculated for each well by constraining the virus control (virus plus cells) as 0% neutralization and the cell-only control (no virus) as 100% neutralization. A 7-point neutralization curve was plotted against serum dilution for each sample, and a four-parameter nonlinear fit was generated using Prism (GraphPad) to calculate the 80% (IC80) inhibitory concentrations.

Biolayer interferometry

Biolayer interferometry was performed with an Octet Red384 (FortéBio). Biotinylated HA protein (A/New Caledonia/20/99, A/Michigan/45/2015, A/Indonesia/05/2005, A/Singapore/1/1957) at 5 μg/mL in assay buffer (PBS +1% BSA), was loaded onto a PBS buffer equilibrated streptavidin-coated biosensor (Sartorius) for 300 s. Biosensors were then equilibrated with assay buffer to remove unbound HA protein to establish a baseline signal for 15 s. Once the baseline was determined, HA protein bound biosensors were assigned to different concentrations of Fab (1600 nM, 400 nM, 100 nM, and 25 nM). After 180 s of association, HA-Fab complexed biosensors were transferred to baseline wells, measuring dissociation for 300 s.

Cell Reports, Volume *43*

Supplemental information

Immune memory shapes human

polyclonal antibody responses to H2N2 vaccination

Yuhe R. Yang, Julianna Han, Hailee R. Perrett, Sara T. Richey, Alesandra J. Rodriguez, Abigail M. Jackson, Rebecca A. Gillespie, Sarah O'Connell, Julie E. Raab, Lauren Y. Cominsky, Ankita Chopde, Masaru Kanekiyo, Katherine V. Houser, Grace L. Chen, Adrian B. McDermott, Sarah F. Andrews, and Andrew B. Ward

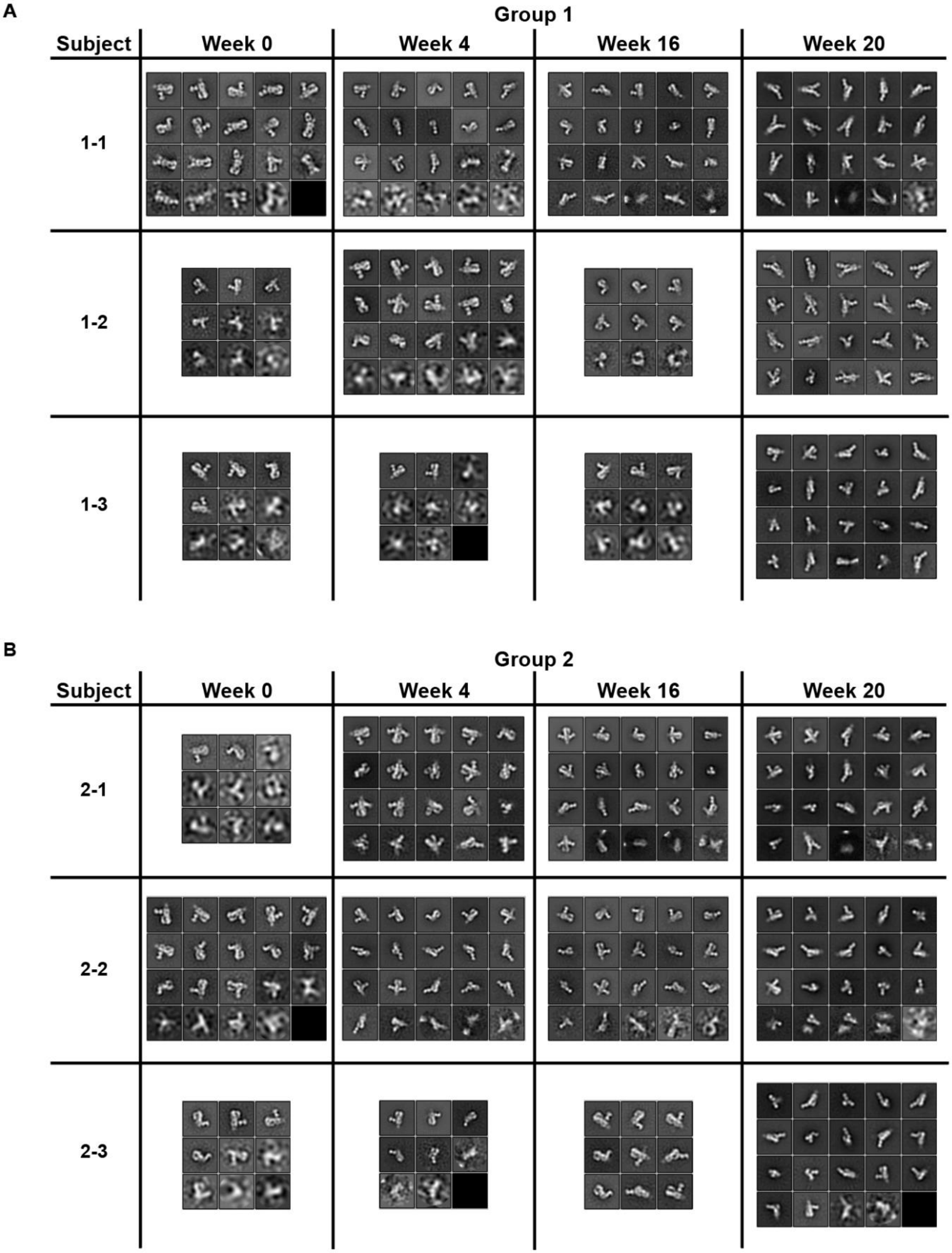

Figure S1: Group 1 and 2 sample 2D classes. Related to Figure 2. Sample 2D classes that make up 3D models shown in Figure 2 for Group 1 (A) and Group 2 (B). Datasets with >7.3k particles are divided into 20 classes while those with <7.3k particles are divided into 9. All 2D classification datasets are shown in order of descending particle count.

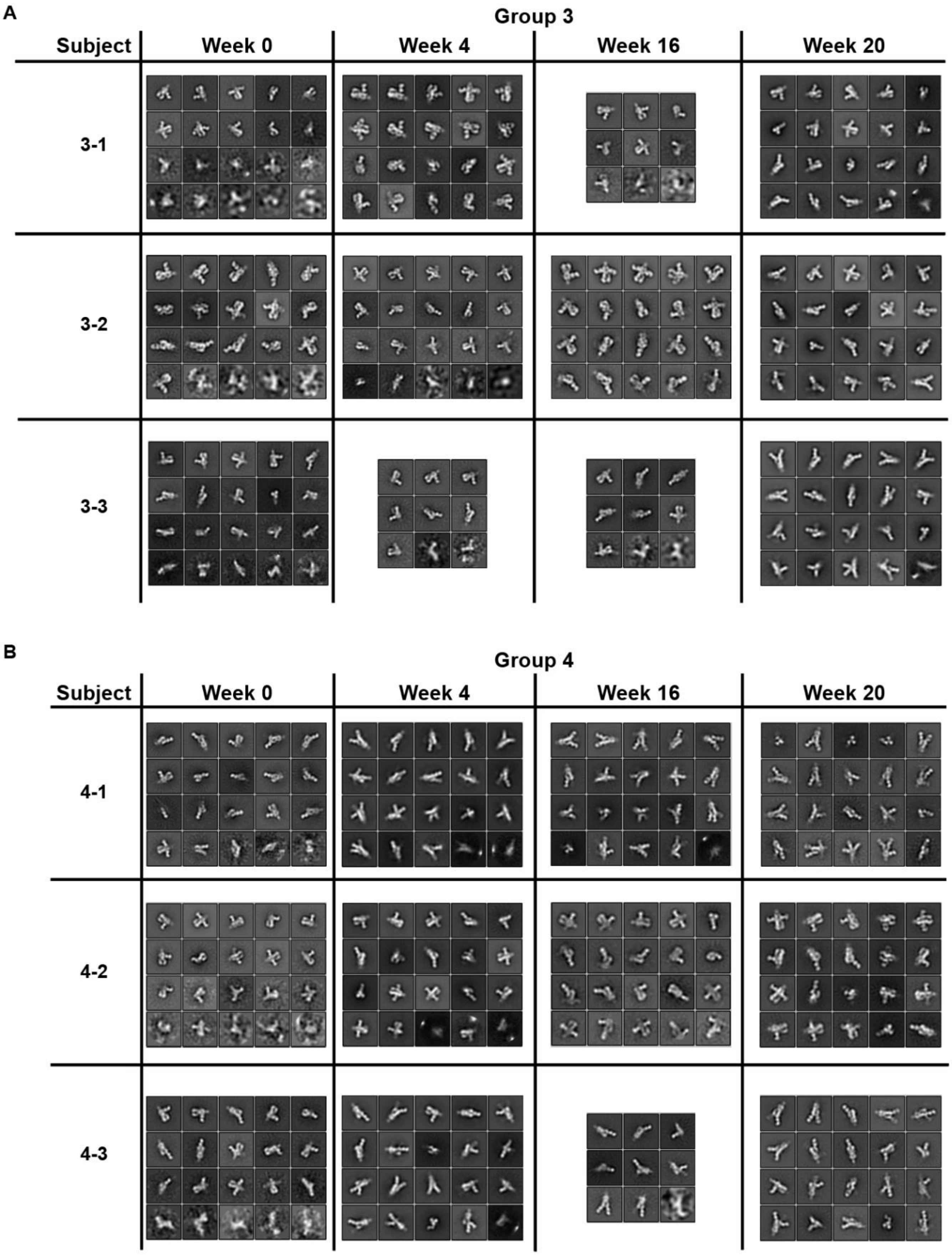

Figure S2: Group 3 and 4 sample 2D classes. Related to Figure 2. Sample 2D classes that make up 3D models shown in Figure 2 for Group 3 (A) and Group 4 (B). Datasets with >7.3k particles are divided into 20 classes while those with <7.3k particles are divided into 9. All 2D classification datasets are shown in order of descending particle count.

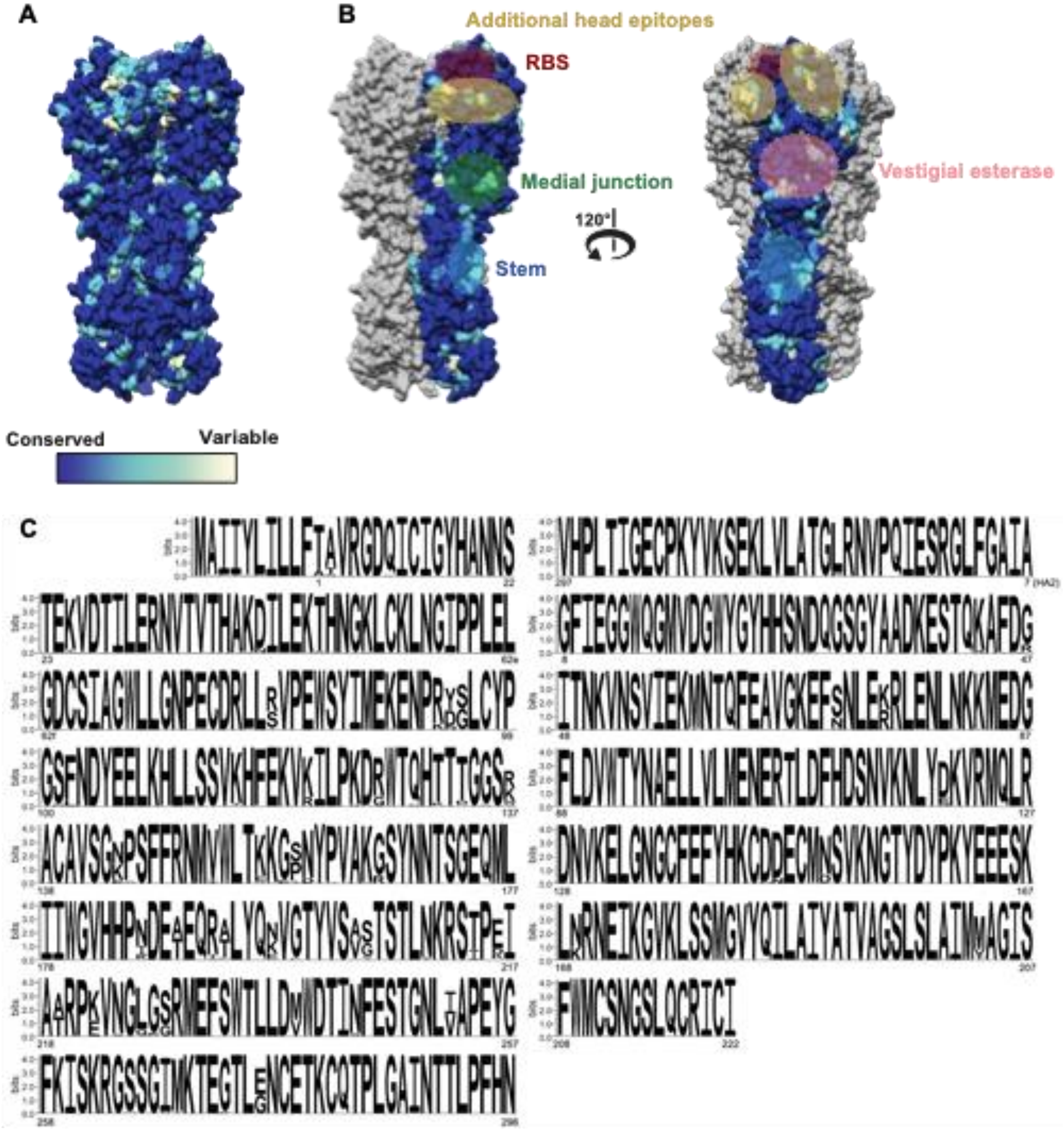

Figure S3: Sequence conservation and antibody footprints on human H2 HA. Related to Figure 2. (A) Sequence variability analysis of 53 human H2 sequences. Sequences for human H2N2 viruses were accessed from GISAID (Global Initiative on Sharing All Influenza Data); duplicate sequences, sequences from different donors of strains with the same geographic location/date, incomplete sequences, and passaged viral experiment sequences were removed, resulting in 53 unique human H2 sequences. Years with sequences represented range from 1857 to 2005. (B) Polyclonal antibody footprints mapped on HA showing H2 variability. (C) Sequence variability analysis of 53 human H2 sequences shown as sequence logo, designed in Librator using WebLogo3.7.

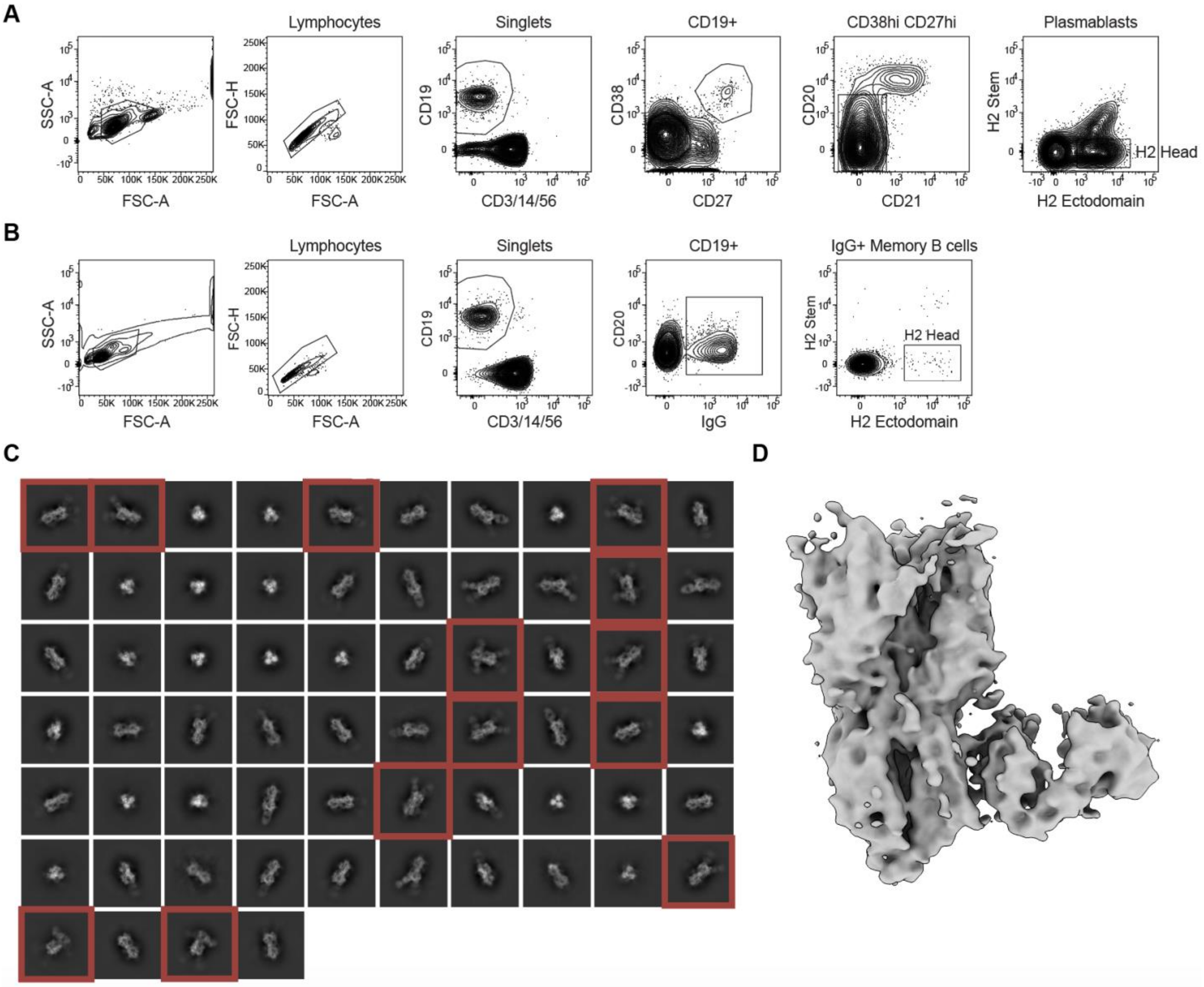

Figure S4: B-cell sorting strategy and polyclonal stem responses observed by cryo-EM. Related to Figure 4 and 5. Flow cytometry gates used to detect and sort H2 HA head-specific B cells from the CD19+ CD3/14/56 (dump)- CD27^{hi} CD38^{hi} CD20^{lo} CD21^{lo} plasmablast (A) or CD19+ CD3/14/56 (dump)- CD20+ IgG+ memory B cell compartment (B). Each plot shows the cell population gated immediately to the left as indicated above each plot. H2 HA head-specific B cells were detected as H2 HA ectodomain+ H2 stem-. (C) 2D classes of HA bound to polyclonal antibodies. Classes featuring pFabs with stem specificities outlined in red. (D) 3D reconstruction of stem-specific pFab.

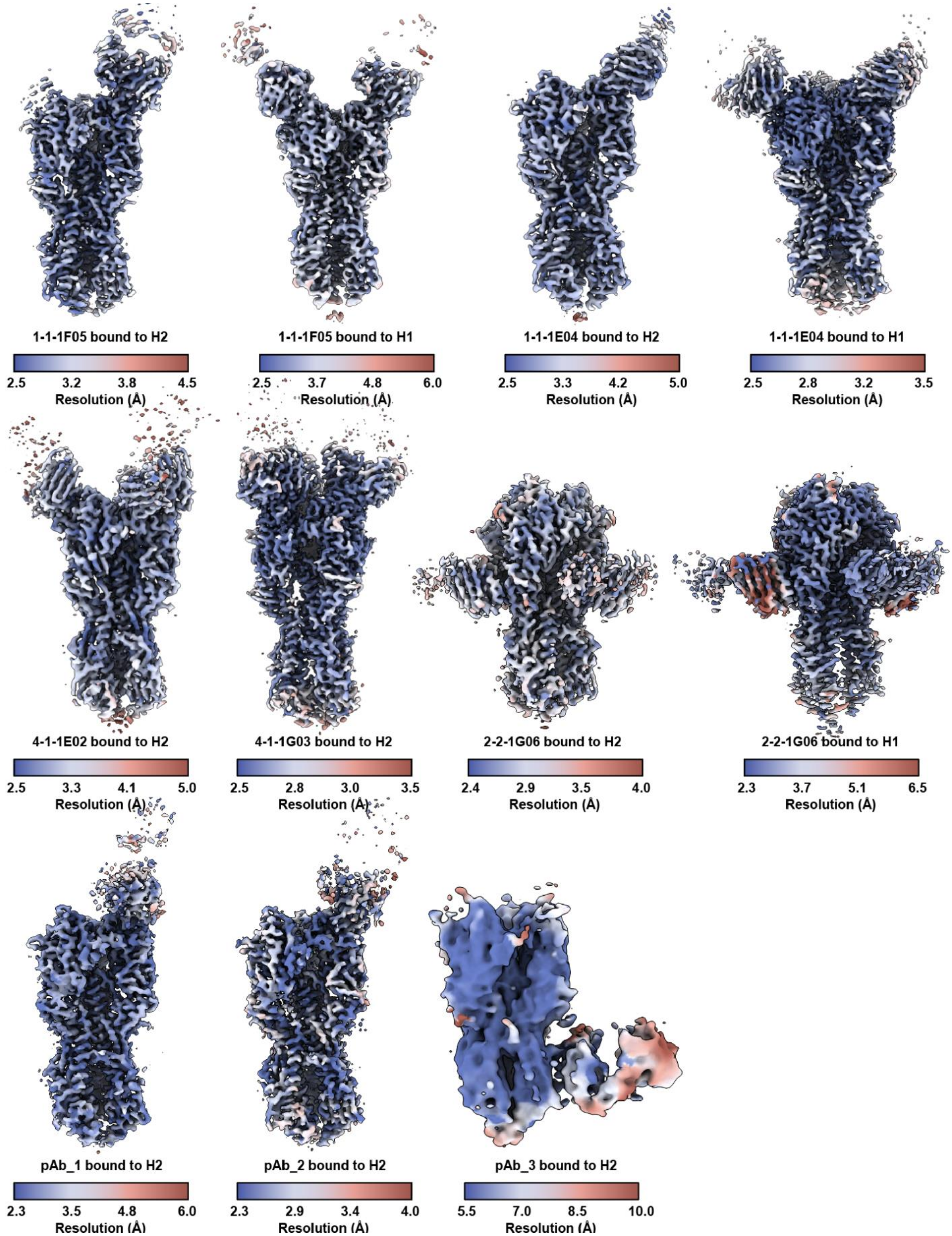

Figure S5: Local resolution plots of EM maps. Related to Figures 5, 6, and 7. Local resolution was calculated according to a 0.143 FSC threshold in cryoSPARC 3.2 and visualized in ChimeraX.

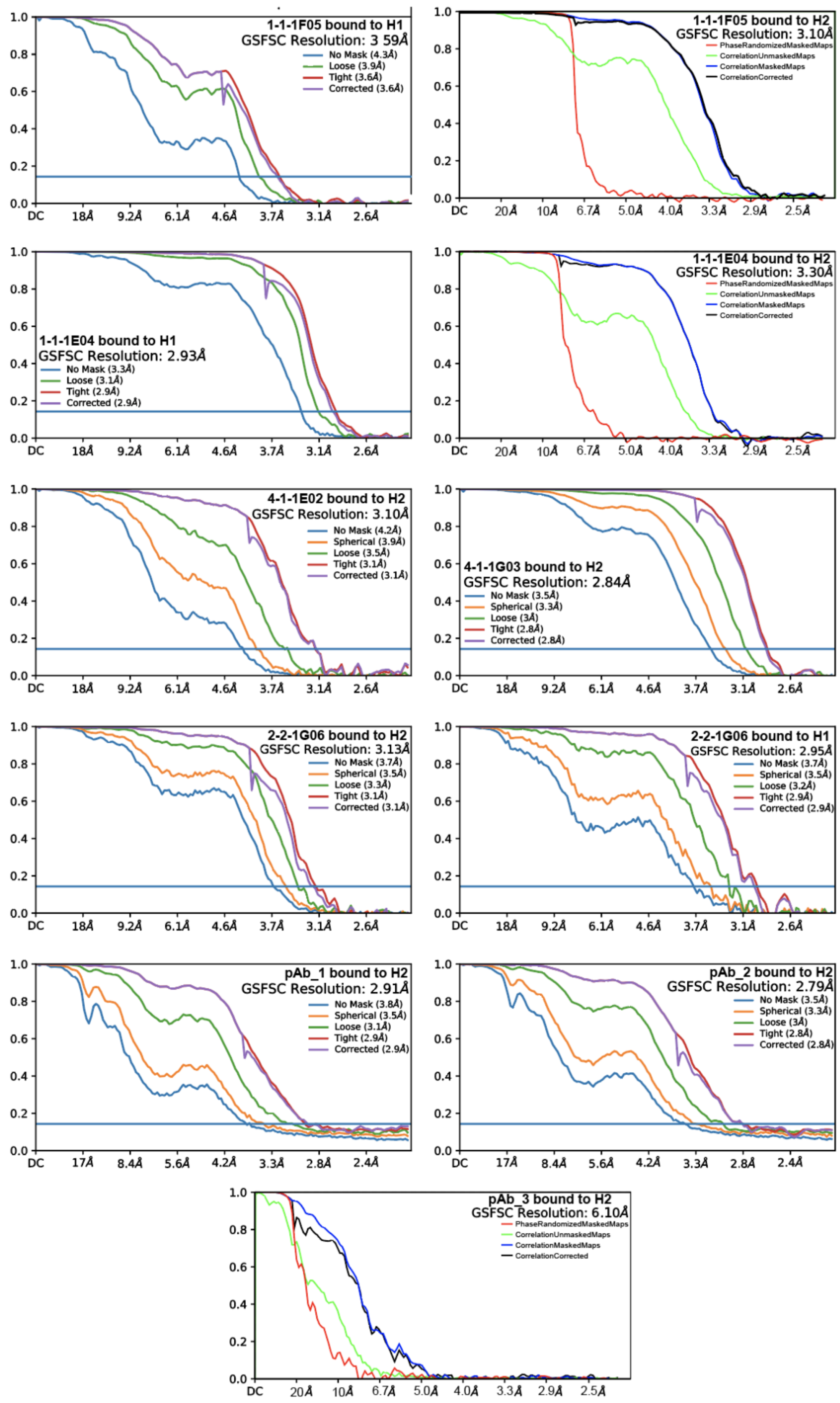

Figure S6: FSC plots for EM maps. Related to Figures 5, 6, and 7. Reported resolutions coincide with an FSC cutoff of 0.143. Plots were generated in cryoSPARC 3.2 or Relion.

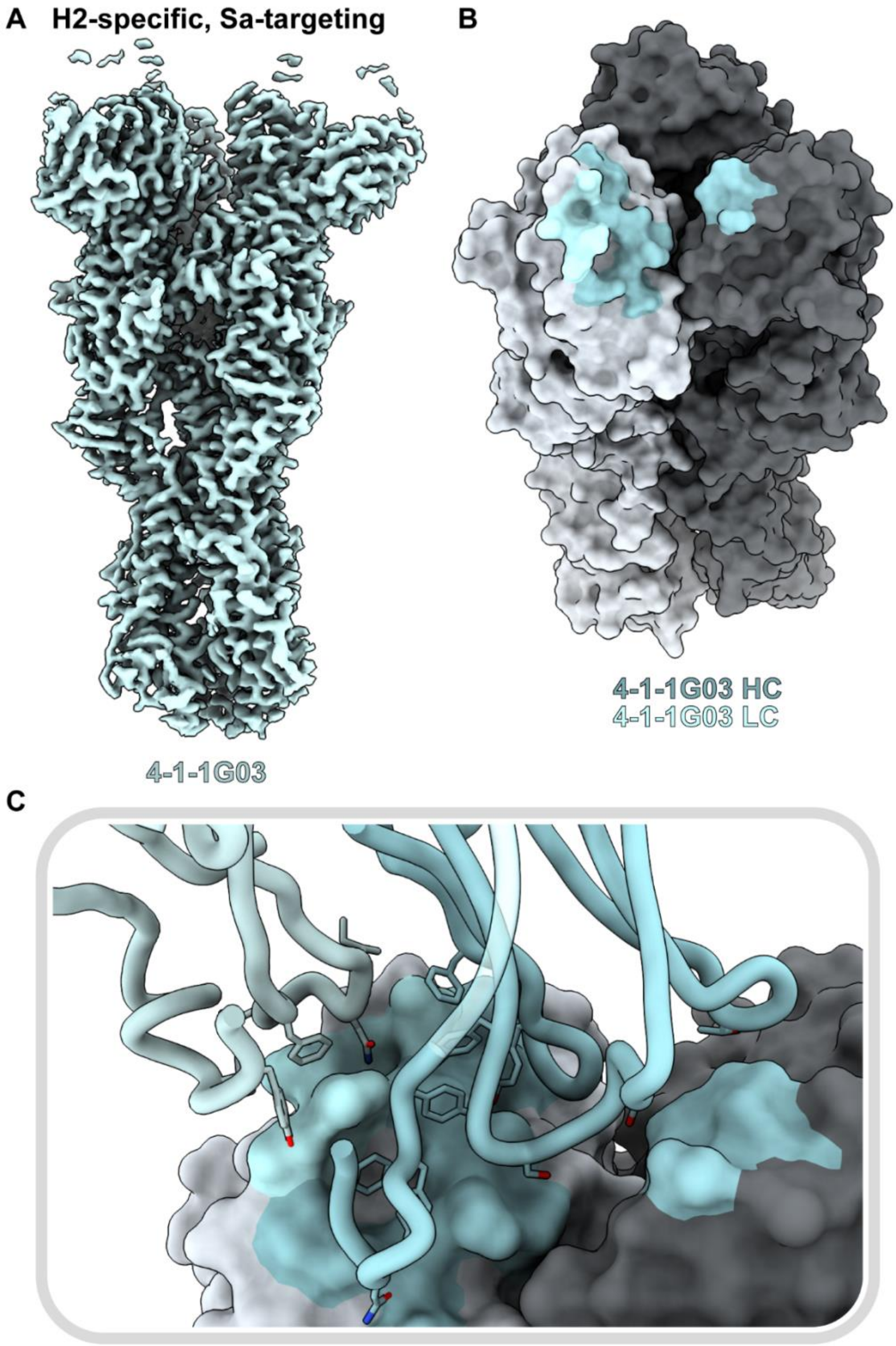

D

	MN IC80 (µg/ml)	
	H1 NC99	H2 Sing/57
1-1-1E04	0.015	>25
1-1-1F05	0.501	>25
4-1-1E02	>25	<0.006
4-1-1G03	>25	0.024

Figure S7: MAb 4-1-1G03 structural characterization. Related to Figure 6. (A) cryoEM density map of 4-1-1G03 Fab complexed with H2. (B) Antibody footprint of 4-1-1G03 colored to indicate heavy and light chain interactions on H2. (C) Antibody loop interactions with the SA epitope with key residues shown. (D) Microneutralization of mAbs against H1 and H2 reporter viruses.

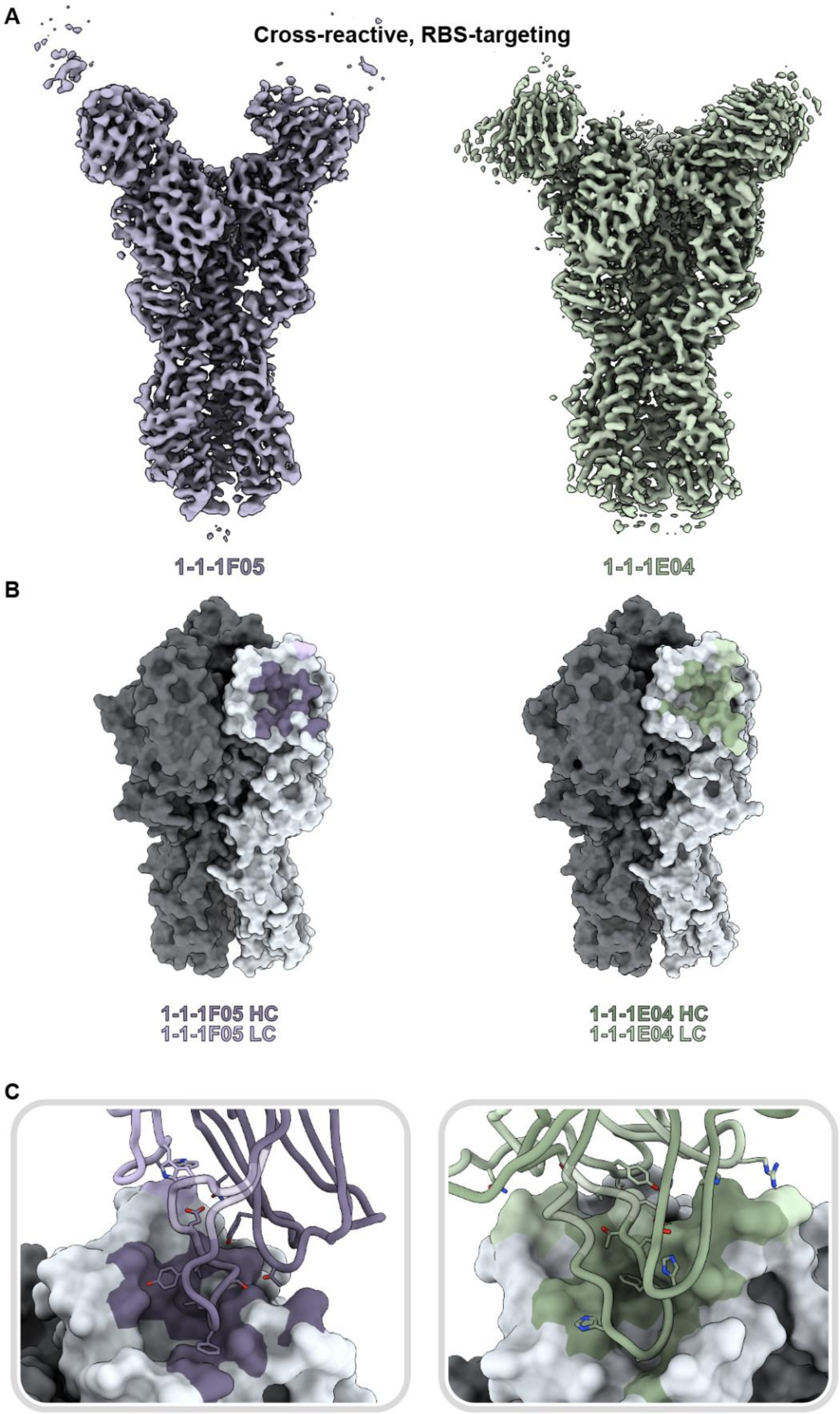

Figure S8: Cross-reactive mAb binding to H1 NC99. Related to Figure 6. (A) Cryo-EM reconstructions of mAbs 1-1-1F05 and 1-1-1E04 bound to H1 NC99. Antibody footprint of 1-1-1F05 and 1-1-1E04 colored to indicate heavy and light chain interactions on H1 NC99. (C) Antibody loop interactions with H1 NC99.

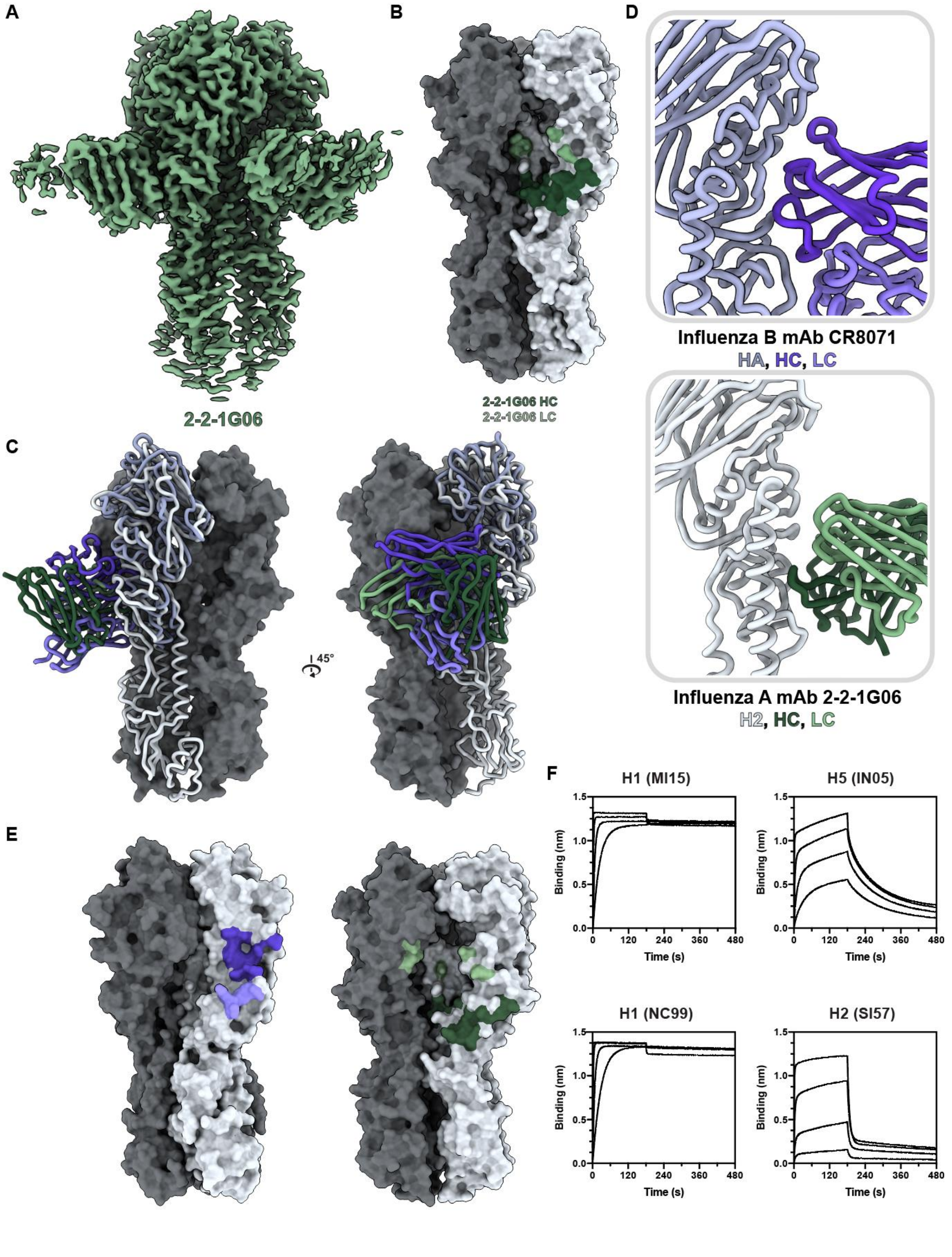

A
2-2-1G06
B
2-2-1G06 HC
2-2-1G06 LC
C
45°
D
Influenza B mAb CR8071
HA, HC, LC
Influenza A mAb 2-2-1G06
H2, HC, LC
E
F
H1 (MI15)
H5 (IN05)
H1 (NC99)
H2 (SI57)
Binding (nm)
Time (s)
0.0
0.5
1.0
1.5
0
120
240
360
480

Figure S9: MAb 2-2-1G06 interaction with H1 and comparison to CR8071 and binding kinetics. Related to Figure 7. (A) cryoEM density map of 2-2-1G06 Fab complexed with H1. (B) Antibody footprint of 2-2-1G06 colored to indicate heavy and light chain interactions on H1. (C & D) Comparison of influenza B mAb CR8071 (purple; PDB 4FQJ) bound to HA (PDBs 4FQJ and 4M44) and 2-2-1G06 (green) bound to H2. (E) Antibody footprints of 2-2-1G06 on H2 and CR8071 on HA (PDBs 4FQJ and 4FQM). (F) BLI sensorgrams indicating immobilized mAb 2-2-1G06 binding to H1, H2, or H5 HAs at concentrations of 3200, 800, 200, and 50 nM.

Table S1: nsEMPEM map and deposition details. Related to Figures 2 and 4.

	EMDB ID	Time point	# particles (composite)	Microscope	Pixel size
**Donor 1-1 (H2)**	EMD-41514	0	7,863	Tecnai TF20	1.77
	EMD-41515	4	11,200	Tecnai Spirit	2.06
	EMD-41516	16	17,496	Tecnai Spirit	2.06
	EMD-41517	20	51,147	Tecnai Spirit	2.06
**Donor 1-2 (H2)**	EMD-41518	0	2,167	Tecnai Spirit	2.06
	EMD-41519	4	9,223	Tecnai TF20	1.77
	EMD-41520	16	3,572	Tecnai Spirit	2.06
	EMD-41521	20	57,429	Tecnai Spirit	2.06
**Donor 1-3 (H2)**	EMD-41522	20	33,782	Tecnai Spirit	2.06
**Donor 2-1 (H2)**	EMD-41523	4	37,664	Tecnai TF20	1.77
	EMD-41524	16	80,050	Tecnai Spirit	2.06
	EMD-41525	20	48,917	Tecnai Spirit	2.06
**Donor 2-2 (H2)**	EMD-41526	0	5,252	Tecnai TF20	1.77
	EMD-41527	4	23,361	Tecnai Spirit	2.06
	EMD-41528	16	12,935	Tecnai Spirit	2.06
	EMD-41529	20	39,714	Tecnai Spirit	2.06
**Donor 2-3 (H2)**	EMD-41530	4	19,649	Tecnai Spirit	2.06
	EMD-41531	16	3,437	Tecnai TF20	1.77
	EMD-41532	20	16,270	Tecnai Spirit	2.06
**Donor 3-1 (H2)**	EMD-41533	0	7,280	Tecnai Spirit	2.06
	EMD-41534	4	27,785	Tecnai TF20	1.77
	EMD-41535	16	31,686	Tecnai Spirit	2.06
	EMD-41536	20	33,852	Tecnai Spirit	2.06
**Donor 3-2 (H2)**	EMD-41537	0	20,800	Tecnai TF20	1.77
	EMD-41538	4	19,649	Tecnai Spirit	2.06
	EMD-41539	16	41,400	Tecnai TF20	1.77
	EMD-41540	20	106,050	Tecnai Spirit	2.06
**Donor 3-3 (H2)**	EMD-41541	0	30,603	Tecnai Spirit	2.06
	EMD-41542	4	27,331	Tecnai Spirit	2.06
	EMD-41543	16	19,700	Tecnai Spirit	2.06
	EMD-41544	20	59,878	Tecnai Spirit	2.06
**Donor 4-1 (H2)**	EMD-41545	0	27,266	Tecnai Spirit	2.06
	EMD-41546	4	54,296	Tecnai Spirit	2.06
	EMD-41547	16	89,300	FEI Talos	1.98
	EMD-41548	20	93,574	Tecnai Spirit	2.06
**Donor 4-2 (H2)**	EMD-41549	0	18,505	Tecnai Spirit	2.06
	EMD-41550	4	50,289	Tecnai Spirit	2.06
	EMD-41551	16	10,340	Tecnai Spirit	2.06
	EMD-41552	20	49,813	Tecnai TF20	1.77
**Donor 4-3 (H2)**	EMD-41553	0	11,809	Tecnai Spirit	2.06
	EMD-41554	4	82,814	Tecnai Spirit	2.06
	EMD-41555	16	14,356	Tecnai Spirit	2.06
	EMD-41556	20	66,790	FEI Talos	1.98
**Donor 1-1 (H1)**	EMD-41557	0	33,500	Tecnai TF20	1.77
	EMD-41558	4	26,600	Tecnai TF20	1.77
	EMD-41559	16	19,300	Tecnai TF20	1.77
	EMD-41560	20	53,500	Tecnai TF20	1.77
**Donor 2-2 (H1)**	EMD-41561	0	34,000	Tecnai TF20	1.77
	EMD-41562	4	88,000	Tecnai TF20	1.77
	EMD-41563	16	65,400	Tecnai TF20	1.77
	EMD-41564	20	45,700	Tecnai TF20	1.77

Table S2: nsEM map and deposition details for monoclonal immune complexes. Related to Figures 4 and 5.

Monoclonal ID	EMDB ID	Breadth	# particles (composite)	Symmetry	Microscope	Pixel size (Å)
**2-2-1E08**	EMD-41683	H2	3,672	C1	Tecnai Spirit	2.06
**2-2-1C06**	EMD-41684	H2	11,091	C1	Tecnai Spirit	2.06
**4-1-1E02**	EMD-41685	H2	25,927	C1	Tecnai Spirit	2.06
**4-1-1G03**	EMD-41686	H2	14,494	C3	Tecnai Spirit	2.06
**1-3-1F08**	EMD-41687	H2	4,424	C1	Tecnai Spirit	2.06
**1-1-1F05**	EMD-41688	cross-reactive	2,741	C1	Tecnai Spirit	2.06
**2-2-1F01**	EMD-41689	cross-reactive	22,641	C1	Tecnai Spirit	2.06
**2-2-1G06**	EMD-41690	cross-reactive	2,651	C1	Tecnai Spirit	2.06
**1-2-189-34**	EMD-41691	cross-reactive	29,435	C3	Tecnai F20	1.77
**1-1-1A09**	EMD-41692	cross-reactive	17020.00	C3	Tecnai F20	1.77
**1-1-2A11**	EMD-41693	cross-reactive	4,231	C3	Tecnai F20	1.77
**1-1-2E05**	EMD-41694	cross-reactive	8,762	C3	Tecnai F20	1.77

Table S3: Cryo-EM map and atomic model refinement. Related to Figures 5, 6, and 7.

	1-1-1F05 bound to H2	1-1-1F05 bound to H1	1-1-1E04 bound to H2	1-1-1E04 bound to H1	4-1-1E02 bound to H2	4-1-1G03 bound to H2	2-2-1G06 bound to H2	2-2-1G06 bound to H1	pAb_1 bound to H2	pAb_2 bound to H2	pAb_3 bound to H2
**Access codes**											
PDB	8TP2	8TP3	8TP4	8TP5	8TP6	8TP7	8TP9	8TPA	N/A	N/A	N/A
EMDB	EMD-41464	EMD-41465	EMD-41466	EMD-41467	EMD-41468	EMD-41469	EMD-41470	EMD-41471	EMD-41472	EMD-41473	EMD-41474
GenBank	BAF48641.1	AAP34324.1	BAF48641.1	AAP34324.1	BAF48641.1	BAF48641.1	BAF48641.1	AAP34324.1	N/A	N/A	N/A
**Data collection and processing**											
Microscope	Talos Arctica	Talos Arctica	Talos Arctica	Talos Arctica	Talos Arctica	Talos Arctica	Talos Arctica	Talos Arctica	Titan Krios	Titan Krios	Talos Arctica
Magnification	36,000	36,000	36,000	36,000	36,000	36,000	36,000	36,000	130,000	130,000	36,000
Voltage (kV)	200	200	200	200	200	200	200	200	300	300	200
Electron exposure (e$^-$/Å^2)	53.5	49.0	49.2	46.2	49.0	49.0	46.2	48.6	49.7	49.7	50.3
Defocus range (μm)	-0.7 to -2	-0.7 to -2	-0.7 to -2	-0.7 to -2	-0.7 to -2	-0.7 to -2	-0.7 to -2	-0.7 to -2	-0.7 to -2	-0.7 to -2	-0.7 to -2
Pixel size (Å)	1.150	1.15	1.150	1.15	1.150	1.150	1.150	1.150	1.045	1.045	1.045
Imposed Symmetry	C1	C1	C1	C3	C3	C3	C3	C3	C1	C1	C1
Final particle number	230,649	105,408	117,851	167,166	103,798	271,581	164,150	124,412	39,631	61,108	17,933
Map resolution (Å)	3.1	3.6	3.3	2.9	3.1	2.8	3.1	3.0	2.9	2.8	6.1
FSC Threshold	0.143	0.143	0.143	0.143	0.143	0.143	0.143	0.143	0.143	0.143	0.143
Map sharpening B-factor (Å^2)	-37.9	-99.7	-40.8	-95.0	-94.9	-105.9	-99.6	-83.7	-56.3	-58.8	-138.4
**Model refinement and validation**											
Total Residues	1697	1720	1697	2223	2153	2163	2157	2172			
Amino-acids	1678	1700	1678	2205	2126	2145	2142	2154			
Carbohydrates	19	20	18	18	27	18	15	18			
RMSD Lengths (Å)	0.020	0.020	0.020	0.021	0.021	0.021	0.020	0.021			
RMSD Angles (°)	1.7	1.7	1.8	1.9	1.8	1.8	1.8	1.8			
**Ramachandran**											
Outliers (%)	0	0	0	0	0	0	0	0	N/A	N/A	N/A
Allowed (%)	2.2	1.7	2.7	1.8	1.2	1.7	1.9	1.3			
Favored (%)	97.8	98.28	97.4	98.21	98.8	98.3	98.1	98.7			
Rotamer outliers (%)	0	0.07	0	0.11	0	0	0	0			
Clash score	1.6	0.8	2.9	1.6	1.9	1.4	2.3	1.5			
Molprobity score	0.94	0.75	1.21	0.91	0.95	0.88	1.00	0.89			
FSC model (0/0.143/0.5)	2.7/2.8/3.1	3.5/3.5/3.9	2.9/3.0/3.3	2.7/2.8/3.1	3.0/3.1/3.3	2.8/2.8/3.0	3.0/3.1/3.3	2.9/2.9/3.2			
EMRinger score	4.2	2.4	4.0	3.4	3.7	4.8	3.5	4.7			
